# Oil‐In‐Water or Water‐In‐Oil Pickering Emulsion: Role of Lignocellulose‐Based Nanoparticle Wettability and Geometry

**DOI:** 10.1002/smsc.70437

**Published:** 2026-11-11

**Authors:** Nissa Nurfajrin Solihat, Muhammad Farooq, Juan José Valle‐Delgado, Elijah Mark Garcia, Kristoffer Meinander, Monika Österberg

**Affiliations:** ^1^ Department of Bioproducts and Biosystems School of Chemical Engineering Aalto University Espoo Finland

**Keywords:** emulsion stability, lignocellulosic nanoparticle, particle geometry, particle wettability, Pickering emulsion

## Abstract

Pickering emulsion behavior is governed by particle wettability, surface chemistry, and geometry, yet stabilization mechanisms for renewable lignocellulosic nanoparticles remain unclear, limiting surfactant‑free design. This study tests hypothesis that tuning the surface energy of lignin nanoparticles shifts emulsion type from oil‐in‐water to water in oil, while combining spherical and fibrillar particles achieves high emulsification capacity and stability through synergy. Influence of spherical lignin nanoparticles (LNPs) and lignin‑containing cellulose nanofibrils (LCNFs) on emulsion type, droplet size, emulsification capacity, and stability is investigated by optical microscopy, dynamic light scattering, conductivity, and field‐emission scanning electron microscopy (FESEM). Unmodified kraft LNPs (K‐LNPs; water contact angle in oil *θ*_o/w_ = 81.6°) form oil‐in‐water emulsions, whereas lauric‐acid‐esterified LNPs (LA‐LNPs; *θ*_o/w_ = 106.4°) favor water‐in‐oil emulsions, confirming wettability‐controlled emulsion type. LNPs generated smaller droplets, but LCNFs stabilized larger oil fractions at lower dosages, demonstrating fibrillar‐geometry efficiency. LCNFs outperform CNFs, emphasizing residual lignin benefits. Synergistic effects of LNPs and LCNFs enhance stability at substantially lower individual concentrations (0.01 wt% LCNFs and 0.02 wt% LNPs) when added together. FESEM reveals that LNPs assembly at oil–water interface, while LCNFs entrap and bridge droplets forming stabilizing network. Tunable geometry and wettability of lignocellulosic nanoparticles enable rational, sustainable Pickering emulsions for diverse applications.

## Graphical abstract

Lignocellulose nanoparticles with tunable wettability and geometry can allow the tailored design of sustainable, surfactant‐free Pickering emulsions. Particle wettability dictates emulsion type, switching between oil‐in‐water and water‐in‐oil. Spherical lignin nanoparticles adsorb at the oil–water interface to create small droplets, while lignin‐containing cellulose nanofibrils bridge droplets into stabilizing networks. Their synergy delivers long‐term stability at ultralow loadings.

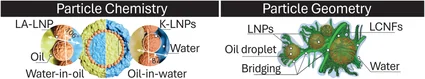

## 1 Introduction

Pickering emulsions are mixtures of typically two immiscible liquids stabilized by solid particles that adsorb at fluid interfaces and form mechanical barriers against droplet coalescence [1, 3]. Unlike molecular surfactants, particle stabilizers irreversibly attached at the oil–water interface because of their high desorption energy, leading to high stability of emulsion [4]. These features make Pickering emulsions attractive for applications where surfactant‐free formulations are desired. Although inorganic particles such as silica, clays, and magnetic nanoparticles have been widely studied as Pickering stabilizers [5], increasing interest in renewable and sustainable materials has motivated the development of lignocellulosic particle stabilizers [6, 7].

Lignocellulosic nanoparticles, including native and modified cellulose nanofibrils (CNFs) and cellulose nanocrystals (CNCs), bacterial cellulose (BC), CNFs with residual lignin (LCNFs), and lignin nanoparticles (LNPs), have gained attention as Pickering emulsifiers due to their unique surface functionality and sustainability [8, 9, 10, 11, 12, 13]. In particular, LNPs possess intrinsic amphiphilicity arising from their aromatic hydroxyl and aliphatic functionalities, which promotes adsorption at oil–water interfaces [9, 14]. However, Pickering stabilization is governed not only by surface chemistry (that dictates particle wettability and surface charge), but also by particle size and morphology. Spherical colloids pack densely and provide isotropic coverage, whereas rods, fibers, and plates introduce orientation‐dependent wetting and line‐tension effects that can stiffen the interfacial film while covering less of the interface [8, 9, 10, 11, 12, 13]. Wettability is especially important because it determines particle attachment energy and the preferred emulsion type. Particles with water contact angles in oil (*θ*_o/w_) below 90° favor oil‐in‐water emulsions, while *θ*_o/w_ above 90° favor water‐in‐oil emulsions [15, 16]. Near‐neutral wettability (*θ*_o/w_ = 90°) gives the highest packing density and strongest interfacial films, enabling stabilization of either emulsion type depending on the water fraction [17].

Despite substantial progress, the independent effects of wettability (surface chemistry) and geometry in lignocellulose‐based Pickering emulsions remain insufficiently resolved. Isolating each variable allows clear structure‐properties relationships to be established, which is important for predictive design of lignocellulose‐based Pickering emulsifiers [18]. In previous studies, more than one parameter often changed simultaneously, such as particle size and charge, or surface chemistry and size [6, 19]. For instance, Pang et al. [19] showed that oil‐in‐water emulsions became more stable as the *θ*_o/w_ of the particle approached 90°, whereas particles with *θ*_o/w_ above 90° favored water‐in‐oil emulsions. However, changes in particle size and surface charge accompanied the wettability variation, making it difficult to assign the observed stability solely to wettability [12]. Similar challenges are observed in recent studies on mixed lignocellulosic particle systems, where multiple properties such as particle chemistry, wettability, charge, size, and shape are varied simultaneously [6, 7, 20], making it difficult to distinguish their individual effects on emulsion stability. CNFs have been reported as co‐stabilizing agent in either LNPs [9], CNCs [7], or TEMPO‐CNFs [20] stabilized emulsion to improve emulsion stability and suppress creaming index. Nevertheless, when both particle types are present simultaneously, their respective contributions to interfacial adsorption, network formation, and droplet stabilization remain unclear.

To address this knowledge gap, we herein systematically elucidate how wettability, surface chemistry, and particle geometry govern the formation and stability of lignocellulose‐based Pickering emulsions. Unlike previous studies where multiple parameters varied simultaneously, this approach decouples individual variables, allowing the effect of each to be assessed independently. To investigate the effect of wettability and surface chemistry, two spherical particles with comparable size and surface charge, denoted as unmodified kraft lignin nanoparticles (K‐LNPs) and lauric acid‐modified lignin nanoparticles (LA‐LNPs), were prepared and characterized. The comparison was extended to fibrillar particles, CNFs and LCNFs, to assess whether differences in surface chemistry and wettability produce similar trends in high‐aspect‐ratio systems. To assess the role of geometry, spherical K‐LNPs and high‐aspect‐ratio LCNFs with comparable surface charge were selected as model particles and studied individually and in combination with concurrent mixing. This design enables evaluation of how LNPs interfacial adsorption, LCNFs fibrillar stabilization, and their combined action contribute to emulsion formation and stability. The results provide mechanistic insight into the rational design of renewable Pickering emulsifiers with tunable interfacial performance.

## 2 Results and Discussion

### 2.1 Characterization of Nanoparticles: Morphology, Wettability, and Surface Chemistry

LNPs and CNFs have been widely used to stabilize Pickering emulsions due to their surface activity and different morphologies. However, it remains unclear how specific particle attributes, such as wettability and geometry, independently and synergistically influence emulsion formation and stability, as the previous works did not decouple the contribution of individual factors [6, 19]. To establish the physicochemical properties of the lignocellulosic nanoparticles intended for Pickering emulsion stabilization, we first characterized their size, surface charge, and wettability. Figure 1a depicts the AFM height images of K‐LNPs and LA‐LNPs, revealing roughly spherical particles with diameters of 30 ± 15 and 22 ± 12 nm, respectively, as measured from AFM height profiles. The corresponding thin films have comparable surface roughness (root mean square average, Rq): 16 ± 1 nm for K‑LNPs and 11 ± 1 nm for LA‑LNPs. In contrast, dynamic light scattering (DLS) measurements in aqueous dispersion yielded hydrodynamic diameters (*D*_h_) of 99 ± 1 nm and 95 ± 2 nm for K‐LNPs and LA‐LNPs, respectively. The higher mean diameters observed in DLS were expected and were probably due to some particle aggregation in suspension, the disproportionate contribution of larger particles to scattering intensity, and the presence of a hydration layer surrounding the nanoparticles [21]. The ζ‐potential of the two LNPs was also similar. K‐LNPs displayed a ζ‐potential of −34 ± 1 mV at pH 3.9, while the ζ‐potential of LA‐LNPs was −40 ± 0.1 mV at pH 6.5 (Figure S2). The pH of the LA‐LNP dispersion was adjusted to pH 6.5 to achieve a surface charge comparable to that of K‐LNPs, thereby minimizing the influence of electrostatic effects and allowing the impact of particle chemistry on Pickering emulsion stabilization to be more directly assessed.

**FIGURE 1:**
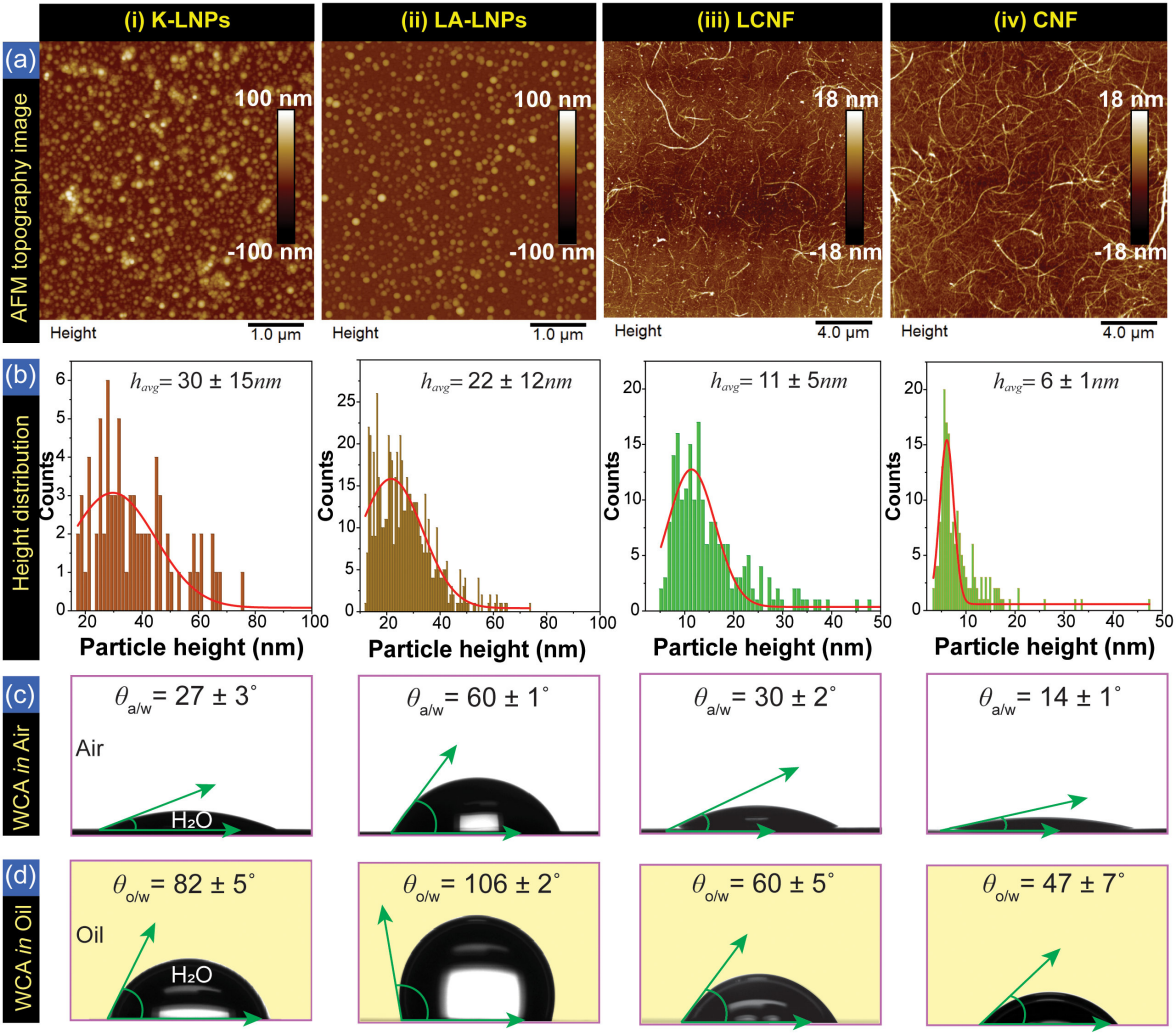
Morphology and wettability of particles: (a) AFM height images; (b) histogram of particle height distribution from AFM images with Gaussian curve fitting; (c) water contact angle in air (*θ*_a/w_); and (d) water contact angle in oil (*θ*_o/w_) of (i) K‐LNPs, (ii) LA‐LNPs, (iii) LCNFs, and (iv) CNFs thin films.

Meanwhile, LCNFs possessed a slightly larger particle height (fibril diameter) distribution (11 ± 5 nm) than CNF (6 ± 1 nm) according to AFM height images (Figure 1a,b). The surface roughness of CNF and LCNFs thin films were quite similar, 3 ± 1 nm. Furthermore, optical microscopy images at larger scale showed the presence of larger fibril bundles in LCNF dispersions, while almost no fibril bundles were observed in CNF dispersion (Figure S3). The length of the fibrils was difficult to assess from the images. The ζ‐potential of LCNFs, −32 ± 10 mV at pH ~ 7**,** was higher compared to CNFs, exhibiting a ζ‐potential of −10 ± 1 mV at pH ~ 7. The more negative ζ‐potential of LCNFs is likely caused by carboxylic or carboxylate groups from residual lignin and hemicelluloses, since LCNFs are obtained from unbleached, lignin containing pulp [22]. The wettability of particles is presented as *θ*_a/w_ and *θ*_o/w_ in Figure 1c,d. K‐LNPs, with *θ*_a/w_ = 26 ± 3° and *θ*_o/w_ = 82 ± 5°, demonstrated a more hydrophilic character than LA‐LNPs, for which *θ*_a/w_ = 60 ± 1° and *θ*_o/w_ = 106 ± 2°. The presence of lignin in LCNFs reduced the hydrophilic character of the cellulose fibrils, as indicated by the slightly higher *θ*_a/w_ (30 ± 2°) and *θ*_o/w_ (60 ± 5°) of LCNFs compared with CNFs (*θ*_a/w_ = 14 ± 1° and *θ*_o/w_ = 47 ± 7°).

To further elucidate differences in the properties and formation mechanisms of K‐LNPs and LA‐LNPs, we performed XPS analysis on bulk lignin powders and LNPs from both K‐lignin and LA‐lignin. When comparing XPS spectra between the bulk lignin powders, LA‐‍lignin was found to have a considerably higher proportion of carbon atoms participating in aliphatic (C─C) and carboxyl (O─C═O) bonds compared with K‐lignin (Figure 2a,b). This confirms that lauric acid chains, containing long alkyl tails as well as carboxyl groups, were present after the esterification between lignin and lauric acid (Figure 2c). Similarly, the relative abundance of C─C in LA‐LNPs was notably higher than in K‐LNPs, while aromatic (ar‐C), carbonyl (C═O), and ether (C─O) components all had lower relative intensities (Table S1, Figure 2b).

**FIGURE 2:**
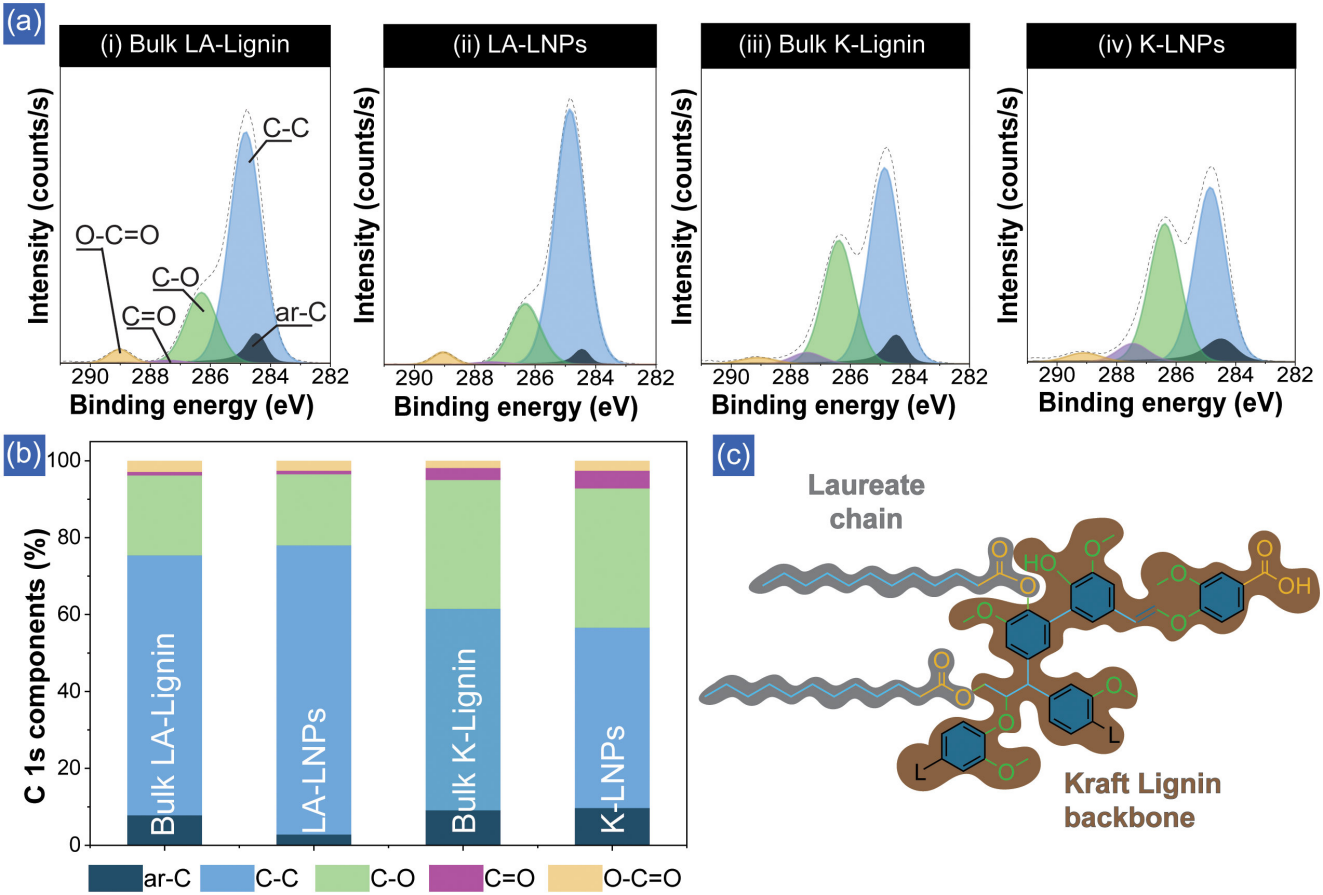
(a) High‐resolution XPS spectra from the C 1s region of (i) bulk LA‐lignin, (ii) LA‐LNPs, (iii) bulk kraft lignin, and (iv) K‐LNPs and (b) relative abundance of various carbon atoms in the C 1s components from XPS spectra. Peaks position is at 284.4 eV (ar‐C), 284.8 eV (C─C), 286.5 eV (C─O), 287.8 eV (C═O), and 288.9 eV (O─C═O). (c) Simplified schematic chemical structure of lignin after modification with lauric acid.

The most important implications, however, arise from a comparison between spectra from the bulk powders and LNPs from either material. When comparing bulk K‐lignin and K‐LNPs in Table S1 and Figure 2a(iii,iv), we observe a considerable decrease in the relative intensity of the C─C component in K‐LNPs (46.9% of the total C 1s intensity) compared to bulk K‐lignin (52.4%), coupled with an increase in the ar‐C component. Contrarily, results for LA‐lignin show an increase in the relative intensity of the C─C component in LA‐LNPs (75.2%) compared with bulk LA‐lignin (67.6%).

In order to fully understand these XPS results, it is important to remember that XPS is a highly surface‐sensitive technique, with a maximum penetration depth of only a few nanometers, and an exponentially higher proportion of the total signal comes from each layer of atoms closer to the surface. This means that high‐resolution XPS spectra are affected by the positioning and orientation of groups, chains, and molecules in the surface layers of the sample studied. When comparing XPS spectra from bulk lignin powders to spectra from LNPs created from essentially the same material, small changes in the relative intensities of different components in the spectra can then be attributed to such changes in the collective positioning and orientation of molecules within the material.

The intensity differences between K‐lignin and K‐LNPs can, therefore, possibly be explained by a certain degree of molecular selectivity during LNP assembly. Initially, more hydrophobic, less soluble molecules, containing a higher proportion of carbon atoms participating in aliphatic C─C bonds, might form the core of the K‐LNP, while smaller molecules, with a relatively higher proportion of aromatic carbon, assemble last at the particle surface. This results in a particle where more hydrophobic groups are contained within the core of the particle, while hydrophilic groups are exposed at the surface of the LNP, much in line with previous findings [23]. Such an assembly will decrease the relative intensity of the C─C component and increase the relative intensity of the ar‐C component, when compared with XPS spectra from the bulk material.

A similar change in intensities might also be expected for LA‐LNPs. Esterification between lignin and lauric acid was done before particle formation, after which nanoparticle formation was done by self‐assembly via solvent shifting. During lignin nanoparticle formation, hydrophobic moieties would then be expected to orient themselves toward the core of the LA‐LNP, while hydrophilic groups are exposed at the surface, because water was used as anti‐solvent. The opposite effect was, however, seen. XPS spectra from LA‐LNPs exhibited an increase in the relative intensity of the C─C component, as compared with bulk LA‐lignin (Figure 2b), suggesting that the hydrophobic alkyl chains of lauric acid are in fact present at higher concentrations on the surface of LA‐LNPs. Similarly, the intensity of the aromatic carbon (ar‐C) was lower in LA‐LNPs than in bulk LA‐lignin, indicating that more aromatic rings of the lignin backbone were positioned or oriented towards the core of the LA‐LNPs.

One possible explanation is that unlike small‐molecule surfactants, which self‐assemble into micelles, steric constraints and limited chain mobility prevent uniform packing of the alkyl chains in LA‐LNPs. Consequently, hydrophobic lauric acid chains may appear exposed at the particle–water interface in spite of their intrinsic low affinity for water because neighboring polar groups influence chain orientation. Moreno et al. [24], who esterified lignin with oleic acid, also speculated that oleate chains were agglomerated on the particle surface. However, they did not confirm that hypothesis with surface sensitive methods such as XPS.

A more detailed explanation of the XPS data is available in the Supporting Information, but in general, our findings show that the difference in surface wettability between K‐LNPs and LA‐LNPs can be attributed to the changes in surface chemistry resulting from lauric acid modification and an exposure of the hydrophobic chains of lauric acid at the surface of LA‐LNPs.

The XPS and wettability findings are in agreement with the surface free energy results of K‐LNPs, LA‐LNPs, LCNFs, and CNFs, calculated from Owens–Wendt–Rabel–Kaelble and acid–base methods. Across methods, the trend was consistent, as summarized in Table S2. The polar component (*γ* ^p^) of LA‑LNPs was markedly lower than for K‑LNPs, LCNFs, and CNFs, indicating that exposed alkyl chains depress the polar contribution of surface free energy and weaken the adhesion to polar liquids. The alkyl chains also increased the hydrophobicity and lowered the total surface free energy of LA‑LNPs (*γ* ^tot^ = 53.8 ± 4.6 mN/m) compared with the K‐LNPs, LCNFs, and CNFs (*γ* ^tot^ in the range of 74–79 mN/m), as determined by Owens–Wendt–Rabel–Kaelble method. Although these particles have different geometries, their surface chemistry is similar. They all have a hydrophilic nature due to abundance of hydroxyl and carboxyl functional groups. Comparing these three nanoparticles, their *γ* ^tot^ ranks as CNF > K‐LNPs > LCNFs, but the differences were small, and all were significantly higher than *γ* ^tot^ of LA‐LNPs. On hydrophobic surfaces, liquids with strong cohesive forces, like water, form higher contact angles, minimizing spread due to lower surface free energy.

Basically, all the particles in this study exhibited higher *γ* ^base^ than *γ* ^acid^. The hydroxyl groups in lignocellulosic structures can act as electron donors, contributing to a higher *γ* ^base^. This is because of their ability to engage in hydrogen bonding [25]. LA‐LNPs had a lower *γ* ^base^ (3.6 ± 1.1 mN/m) compared with K‐LNPs, LCNFs, and CNFs (≥5.7 mN/m), which indicated a lower hydroxyl group content in LA‐LNPs due to the esterification with lauric acid, which occurs at the hydroxyl groups of lignin [26]. As the surface becomes more hydrophobic, it tends to repel polar substances, which aligns with weaker electron‐donating interactions and inherently decreased *γ* ^base^ [27]. Given that most biopolymers are generally basic, the larger calculated *γ* ^base^ compared with *γ* ^acid^ was expected [28].

### 2.2 Effect of Particle Wettability on Pickering Emulsion Properties

The degree of wettability of the particles significantly dictates their affinity for either the oil or the water phase, thus influencing how effectively they can stabilize emulsions. Prior studies have shown that lignin particle wettability influences droplet size and stability of emulsion [19, 29], but studies on the effect of wettability on tailoring the type of emulsion are limited. To address this knowledge gap, we explored if we could control the type of emulsion simply by tuning particle wettability, as well as if the W:O ratio had any effect on the emulsion type. To do this, we prepared emulsions with 1 wt% LNPs content and varied the W:O ratio from 2:1 to 1:2, with hexadecane as the oil phase (Figure 3a,b). According to the *θ*_o/w_ results, we expected K‐LNPs to form oil‐in‐water emulsions (*θ*_o/w_ = 82 ± 5°) and LA‐LNPs to create water‐in‐oil emulsions (*θ*_o/w_ = 106 ± 2°) (Figure 1d). This hypothesis was confirmed with electrical conductivity measurements of the emulsions and optical microscopy images of the droplets (Figures 3a–c, S4), as well as by the fact that emulsions with K‐LNPs could be diluted with water, but emulsions with LA‐LNPs could not (indicating that the continuous phase in the latter was oil).

**FIGURE 3:**
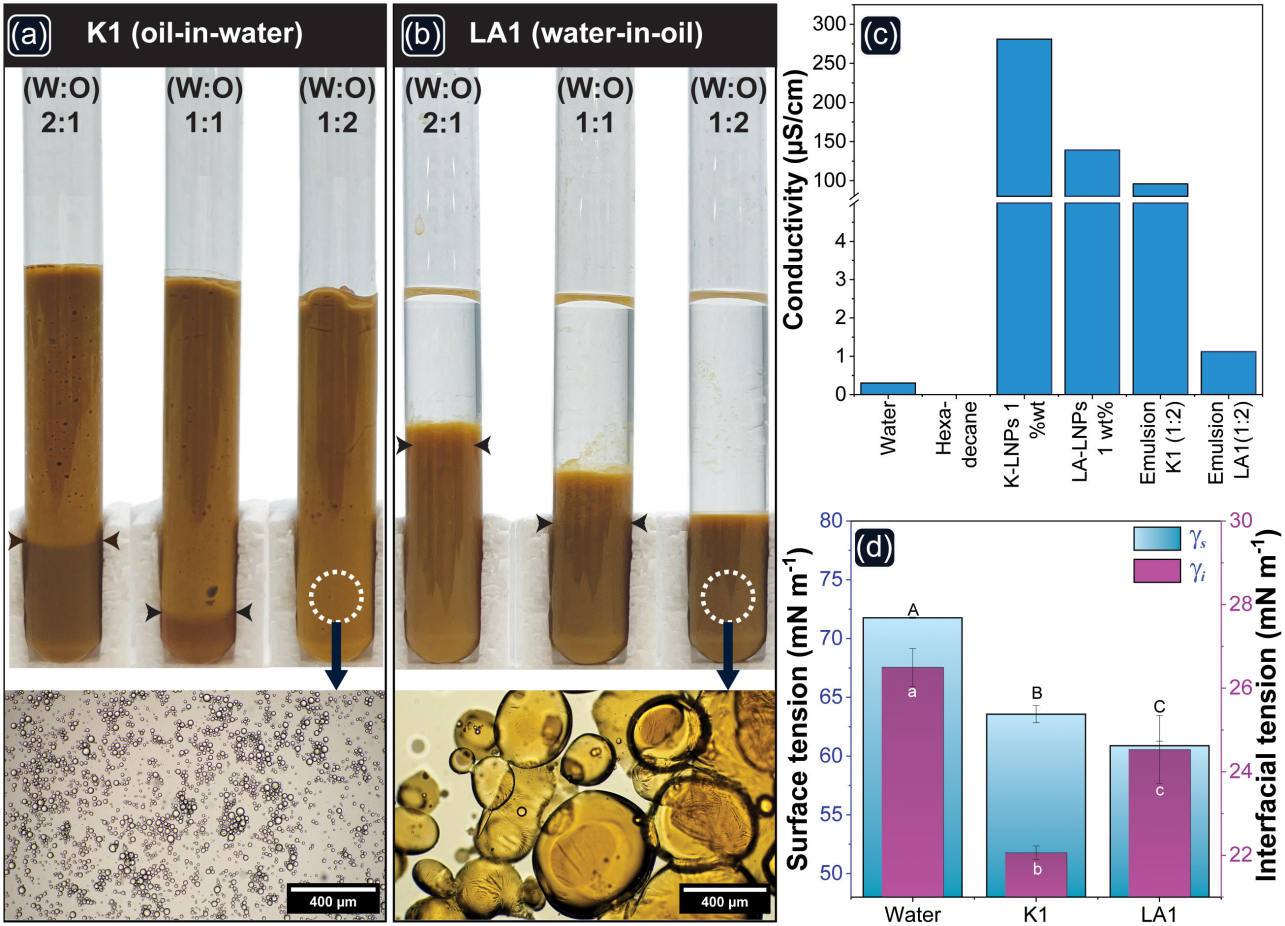
Visual investigation of (a) oil‐in‐water emulsion from 1 wt% K‐LNPs and (b) water‐in‐oil emulsion from 1 wt% LA‐LNPs with different W:O ratio (2:1, 1:1, 1:2) and their droplet images. Black arrow symbols indicate the interface between emulsion and aqueous nanoparticle dispersion. (c) Conductivity of water, oil, particles, and emulsions with W:O ratio 1:2. (d) Surface and interfacial tension of 1 wt% K‐LNPs and 1 wt% LA‐LNPs. Note: For surface and interfacial tension graph (d), the distinct letters within the same bar category indicate significant differences at a 95% confidence level.

For the electrical conductivity measurements, we selected 1:2 W:O emulsions as representative samples because of the well‐defined emulsion volumes. Emulsions prepared with LA‐LNPs had a very low electrical conductivity (1 µS cm^−1^), comparable to that of hexadecane (0 µS cm^−1^), confirming that oil was the continuous phase in these emulsions (Figure 3c). In contrast, the emulsion stabilized with K‐LNPs exhibited high conductivity (96 µS cm^−1^), indicating that water containing LNPs was the continuous phase in the emulsion. This high conductivity is attributed to the presence of ionic species like LNPs and counterions in the continuous medium, thereby confirming the oil‐in‐water emulsion structure. The lower conductivity value of the K‐LNPs emulsion compared to 1 wt% aqueous dispersion of K‐LNPs without any oil phase (281 µS cm^−1^) is due to the fact that many K‐LNPs were adsorbed at the oil droplet interfaces, reducing the free particle and counterion population in the continuous phase.

Optical microscopy images also confirm the different emulsion types formed by K‐LNPs and LA‐LNPs. The brown color of droplets of water‐in‐oil LA1 emulsions observed in the optical images of Figures 3b and S4c,d was due to the excess of lignin nanoparticles dispersed in the water phase, while the oil might be detected as a continuous phase. The brown color of droplets was detected in the droplets of all LA‐LNPs series at different W:O ratios (Figure S4). The brown color was not clearly visible in the continuous phase of the optical images of oil‐in‐water K1 emulsions (Figures 3a and S4a,b) because these samples were diluted with water 10–50 times before inspecting them with the optical microscope. In addition, the water‐in‐oil droplets had a more irregular shape (Figure 3b) compared with oil‐in‐water droplets, which were spherical (Figure 3a). This is in line with previous observations of water‐in‐oil emulsions [30], and it might be due to the higher viscosity and lower density of oil as the continuous phase [30, 31].

On the other hand, our finding indicate that the W:O ratio does not affect the emulsion type (at least in the range from 1:2 to 2:1) when K‐LNPs and LA‐LNPs are used as emulsion stabilizers (Figure S4). Thus, the most important factor deciding the emulsion type was in our case the wetting properties of the particles, not the ratio between oil and water. The comparison of droplets sizes for emulsions stabilized by K‐LNPs and LA‐LNPs in different W:O ratios is summarized in Table S3. In general, K‐LNPs series at different W:O ratios obtained significantly smaller droplet size (in the range of 8–12 µm) compared with LA‐LNPs series (210–370 µm). Larger droplet size from emulsion stabilized by LA‐LNPs correlates with higher *γ* *_i_* of 1 wt% LA‐LNPs (24.5 mN m^−1^) compared with 1 wt% K‐LNPs (22.1 mN m^−1^), presented in Figure 3d. The higher *γ* *_i_* indicates a lower tendency of LA‐LNPs to adsorb at the oil–water interface in comparison with K‐LNPs [10]. Besides, LA‐LNPs presented lower emulsification efficiency compared with the K‑LNPs, as evidenced by the presence of an excess oil phase for LA‑LNPs. This aligns with their wettability: K‐LNPs exhibited *θ*_o/w_ ≈ 82°, closer to 90° than LA‐LNPs (106°), conferring stronger interfacial adsorption. Interestingly, LA‐LNPs at 1 wt% concentration were more effective in reducing air‐water surface tension compared with K‐LNPs at the same concentration, which suggests that LA‐LNPs may be more efficient than K‐LNPs at stabilizing Pickering foams. All the lignin nanoparticle stabilized emulsions were stable for at least a week, as shown by a similar droplet distribution in Figure S4a–d and droplet size in Table S3. Besides, the excess nanoparticle dispersion at the bottom of the tube in both K1 and LA1 with different W:O ratio exhibited negligible droplet formation on the micrometer scale (Figure S4e).

Inspired by the strong impact of spherical LNP particle wetting properties on emulsion type, we next explored the effect of wettability of fibrillar particles on emulsion properties by comparing LCNFs and CNFs. As shown in Figure 1c,d, CNFs were more hydrophilic than LCNFs, which affected the emulsification properties, as presented in Figure 4a. At the same particle concentration (0.1 wt%) and W:O ratio of 2:1, LCNFs were able to emulsify all the oil in the system. In contrast, an excess of non‐emulsified oil was detected in CN0.1, demonstrating that LCNFs were better Pickering stabilizers than CNFs. Both LCNFs and CNFs produced oil‐in‐water emulsions, as expected based on their low three‐phase contact angle in oil: *θ*_o/w_ < 90°.

**FIGURE 4:**
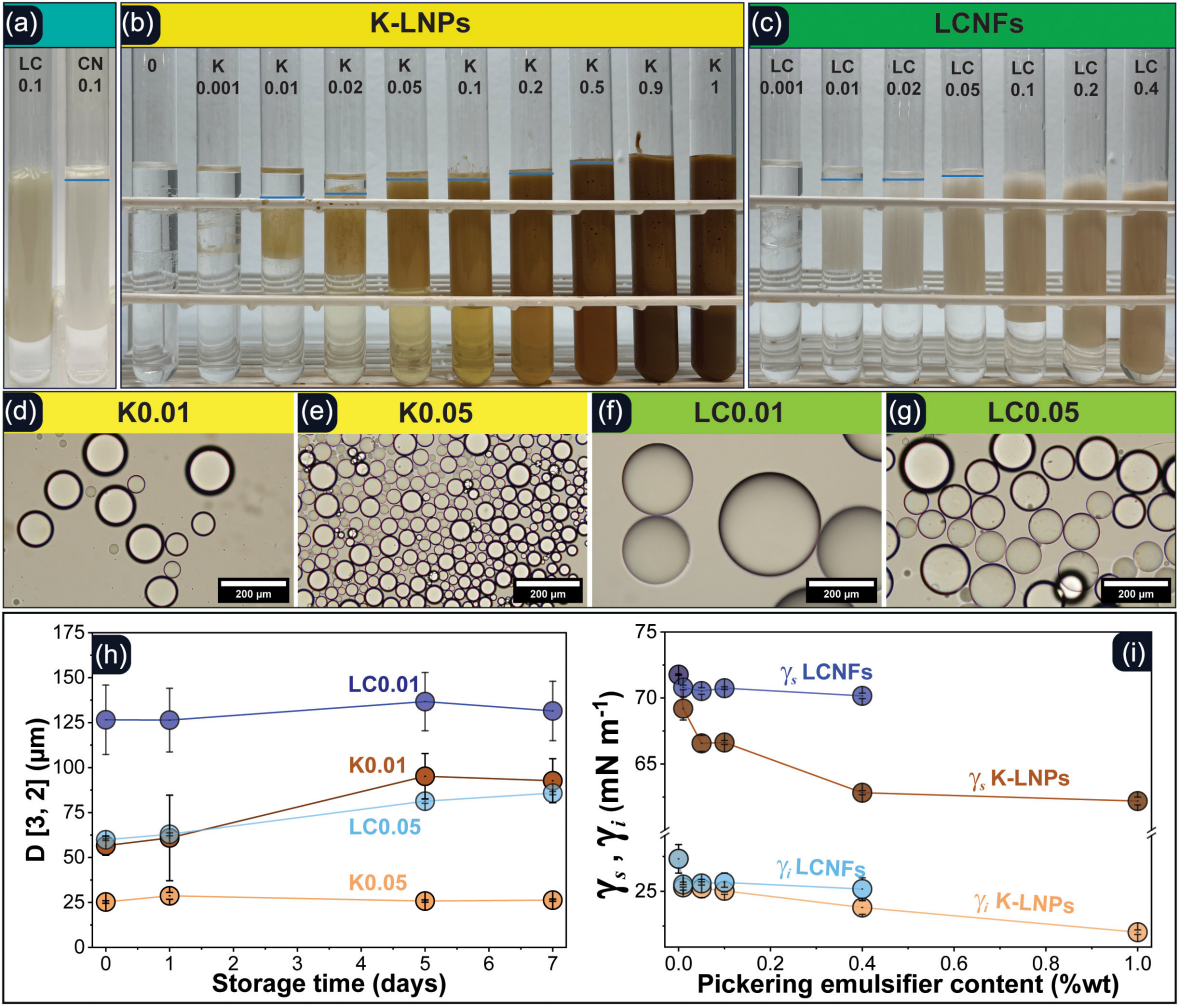
Effect of K‐LNPs, LCNFs, and CNFs content on Pickering emulsion stability, droplet size, surface (*γ*_s_) and interfacial (*γ* *_i_*) tension in single emulsifier systems. (a) Visual assessment of emulsion stabilized by 0.1 wt% LCNFs vs. 0.1 wt% CNFs (b) 0–1 wt% K‐LNPs, and (c) 0.001–0.4 wt% LCNF after 1 day storage. Optical micrographs of emulsion droplets containing (d) 0.01 wt% K‐LNPs, (e) 0.05 wt% K‐LNPs, (f) 0.01 wt% LCNF, and (g) 0.05 wt% LCNF after 1 day storage. (h) Droplet size of emulsion after a week of storage, and (i) *γ*_s_ and *γ* *_i_* as a function of concentration of K‐LNPs and LCNFs, respectively. Blue lines in (a)–(c) identify the oil and emulsion interface.

To sum up, the wettability of nanoparticles, both spherical and fibrillar, had a dominant role over other factors like water‐oil ratio on the formed emulsion type. Even at W:O ratio 1:2, particles having *θ*_o/w_ < 90° still produced oil‐in‐water emulsion. In contrast, particles having *θ*_o/w_ > 90° produced water‐in‐oil emulsions at W:O ratio 1:2. The emulsion stabilization mechanism of fibrils may also hamper the possibility of forming water‐in‐oil emulsions as long as the fibrils are dispersed in the water phase, as will be discussed further in the next section. Optimizing W:O ratio with higher content of hydrophobic particle is worth studying in the future to produce water‐in‐oil emulsions without an excess oil phase.

### 2.3 Effect of Particle Geometry on Emulsion Stabilization Mechanism

Beyond wettability, the geometry of a particle fundamentally governs its orientation and packing behavior at the oil–water interface, directly influencing the stability mechanism. Agustin et al. [6] reported that emulsion stabilized by LNPs had a greater ability to emulsify oil than TEMPO‐CNFs. However, those two particles have different charges and wetting properties, which may also have contributed to the stability of the emulsion, as shown in Section 2.2 and also reported by Tomita et al. [12]. To pinpoint the specific effect of particle geometry on emulsion formation and stability, we selected spherical K‐LNPs and fibrillar LCNFs with similar ζ‐potential and wettability for comparison. In this way, the impact of surface charge or differences in wetting properties on the Pickering emulsion characteristics was minimized. The W:O ratio was kept constant at 2:1 across all emulsion series.

As expected, increasing the Pickering emulsifier content from 0.001 to 1 wt% for LNPs and from 0.001 to 0.4 wt% for LCNF significantly reduced the volume of non‐emulsified oil in the system, as shown in Figure 4b,c and Table S4. This improvement was a consequence of the greater availability of particles to adsorb at the oil–water interface, enabling more efficient emulsification and incorporation of the oil phase into stabilized droplets. However, a higher LNP content was required to fully emulsify the oil phase compared to LCNFs (Figure 4b,c). Indeed, 0.1 wt% LCNFs were able to emulsify all the oil volume, as indicated by the absence of observable excess of oil fraction in the emulsion, whereas more than 0.5 wt% K‐LNPs was required to obtain similar emulsification efficiency (Figure 4b,c and Table S4). In spite of having similar surface charges, these differences underscore the impact of other factors like the geometry of particle on emulsion stability. Furthermore, higher Pickering emulsifier content (LNPs or LCNFs) resulted in smaller droplet sizes (Figures 4d–g, S5), as a larger total interfacial area could be stabilized when the particle concentration increased, allowing the formation and preservation of smaller droplets during homogenization [32]. The emulsions and the droplet size were stable for at least 7 days (Figures 4h, S5 and S6), indicating that the particles irreversibly adsorbed at the oil–water interface and prevented oil droplet coalescence via steric and electrical double layer repulsions [4]. But what was especially interesting was that LNPs produced significantly smaller droplet sizes compared to LCNFs at equivalent concentrations (Figure 4d–g). Both the different effects on droplet size and the different concentrations of particles needed for complete emulsification of the oil could be related to the different geometries of LNPs and LCNFs. Spherical particles can pack more easily at the oil–water or air‐water interfaces than long fibrils, resulting in lower air‐water surface tension (*γ*_s_) and oil–water interfacial tension (*γ* *_i_*), as depicted in Figure 4i. K‐LNPs had stronger affinity to the air‐water and oil–water interfaces than LCNFs, as indicated by a smaller emulsion droplet size stabilized with LNPs compared to LCNFs (Figure 4h). Since smaller oil droplets were formed with K‐LNPs, more K‐LNPs were needed to fully emulsify the oil because the total oil–water interfacial area increased with decreasing droplets size. In contrast, the fibrillar LCNFs, with a higher aspect ratio compared to spherical particles, provided more effective steric stabilization and fibril network formation in the water phase. This network results in a steric hindrance, preventing coalescence of oil droplets [6]. Thus, these two different geometries of particles had different stabilization mechanisms: LNPs excel at creating fine emulsions by packed arrangement of nanoparticles around the oil droplets, which required higher concentrations for complete stabilization of the whole oil content; in contrast, LCNFs provide robust mechanical stability mainly through fibrillar‐network stabilization at lower concentrations, despite forming larger droplets. Our results confirmed that particle geometry had an effect on emulsion formation and stabilization. A similar trend was reported for CNCs with varying aspect ratios by Kalashnikova et al. [33], who observed that the emulsified oil fraction increased with CNC aspect ratio, but smaller oil droplets were obtained with low aspect ratio CNCs. In addition to aspect ratio, fibril bending energy may also contribute to emulsion formation and stabilization [34]. In fact, fibril flexibility is expected to have opposite effects on each of the two mechanisms for emulsion stabilization discussed above. In principle, fibrils could cover the surface of oil droplets more efficiently as their flexibility increases, but higher flexibility also implies the formation of less strong fibril networks in the continuous phase. The results from Lu et al. [34] indicated that the detrimental effect of fibril flexibility on the mechanical strength of the fibril networks seemed to be the dominant factor. Indeed, they observed that flexible bacterial nanocellulose, with high aspect ratio, showed poor emulsification capacity compared with semi‐flexible CNFs, at the range of concentrations studied. Together, these studies indicate that both aspect ratio and fibril flexibility can influence emulsification and that their relative contribution may depend on the system.

### 2.4 Combination of LNPs and LCNFs: Synergistic Effect for Pickering Emulsion Stabilization

Encouraged by the different stabilization mechanisms of LCNFs and LNPs, our next step was to explore possible synergies when using a combination of the two particles. However, combining more than one emulsifier makes it difficult to identify the most important parameters for emulsion stabilization as well as the stabilization mechanism. Some reports have explained the synergistic effect of CNFs added into a LNPs‐stabilized emulsion to obtain a stable emulsion with 0% creaming index [6, 9]. However, there is still a lack of a basic understanding of co‐stabilization. For example, when these two particles are concurrently mixed, which one adsorbs at the interface first, and in what order do they assemble? Here, we addressed this knowledge gap by investigating the synergistic effect of the combination of LNPs with LCNFs in a one‐pot system with well characterized particles. We analyzed the characteristics of Pickering emulsions formed when keeping the concentration of K‐LNPs constant at 0.02 wt%, while the LCNFs content varied from 0.01 to 0.05 wt%. This emulsifier content was selected to isolate the synergistic effect between K‐LNPs and LCNFs, rather than to reflect a mere increase in total solids content. As demonstrated in Section 2.3, the emulsion containing 0.03 wt% of either particle type alone was not enough to emulsify the full volume of oil fraction. As shown in Figure 5a, the combination of K‐LNPs at 0.02 wt% and LCNFs at 0.01 wt% successfully produced a stable emulsion with no observable oil phase separation. This demonstrates that even a minimal increase in LCNF concentration was sufficient to effectively emulsify the excess of oil phase. Additionally, the droplet size of that emulsion was smaller compared with the emulsion containing only 0.01 wt% LCNFs, as depicted in Figure 5b. However, it was larger than the droplet size observed in the emulsion containing only 0.02 wt% K‐LNPs. For instance, the droplet size of emulsion after 1 day of storage according to DLS was as follows: 60 ± 19 µm for emulsion containing only 0.02 wt% K‐LNPs (K0.02); 126 ± 18 µm for emulsion containing only 0.01 wt% LCNFs (LC0.01); and 89 ± 4 µm for combination 0.02 wt% K‐LNPs and 0.01 wt% LCNFs (K0.02 + LC0.01). LNPs were more effective in obtaining smaller droplets than LCNFs because LNPs stabilize the oil by creating compact particle shells around oil droplets while LCNFs mainly promote stability by fibrillar network formation. When both K‐LNPs and LCNFs were combined, the droplet size was between the sizes obtained with each stabilizer alone. This could be explained by a competition between K‐LNPs and LCNFs to adsorb at the oil–water interface. The insignificant differences in *γ* *_i_* for K0.02 and LC0.01 corroborate this hypothesis. In addition, LCNFs may hinder, to some extent, the diffusion of K‐LNPs towards oil–water interfaces. Hence, both mechanisms would lead to oil droplet sizes bigger than those with only K‐LNPs. Although *γ* *_i_* of K0.02 and LC0.01 were quite similar, their combination was beneficial due to their different morphology. When LNPs and LCNFs are concurrently mixed, the elongated fibrils of LCNFs either co‐adsorbed at the interface to fill gaps in LNPs‐stabilized droplets or form a 3D network in the aqueous phase to trap and immobilize LNPs‐stabilized droplets. This hypothesis was supported by FESEM and Cryo‐FESEM images of the droplets (Figure 6).

**FIGURE 5:**
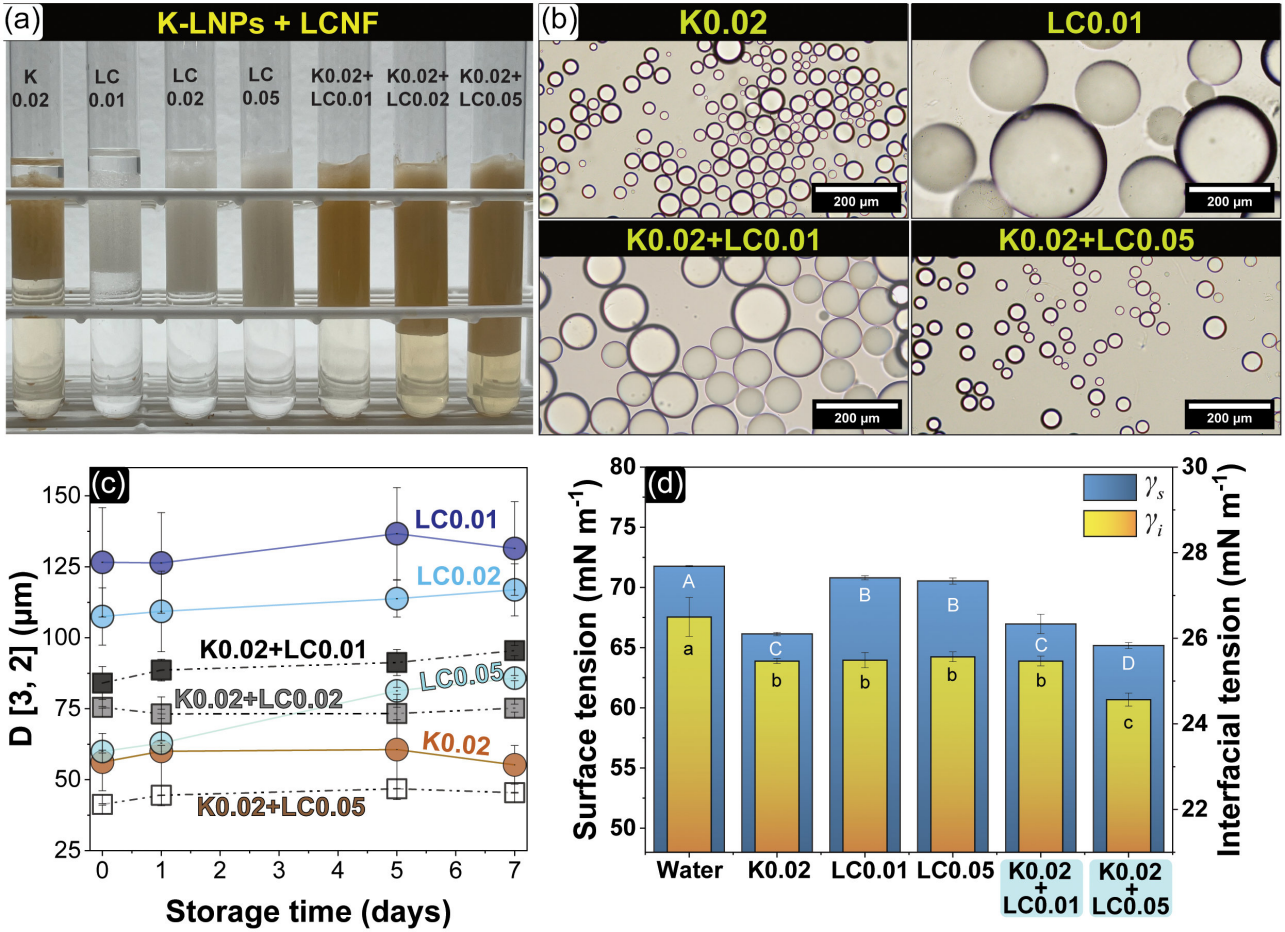
Simultaneous effect of combination of K‐LNPs and LCNF with different content on Pickering emulsion stability, surface and interfacial tension, droplet size, and emulsion micrograph. (a) Visual appearance, (b) droplet micrograph of emulsion containing 0.02 wt% K‐LNPs, 0.01 wt% LCNF, combination of 0.02 wt% K‐LNPs + 0.01 wt% LCNF, and combination of 0.02 wt% K‐LNPs + 0.05 wt% LCNF. (c) droplets size by Mastersizer of emulsion containing only 0.02 wt% K‐LNPs, only LCNF with different content (0.01–0.05 wt%), and in the combination of 0.02 wt% K‐LNPs with different content of LCNF (0.01–0.05 wt%) after 1 day emulsification. (d) *γ*_s_ and *γ* *_i_* of only K‐LNPs, only LCNF, and combination of K‐LNPs with different content of LCNF. Note: For surface and interfacial tension in graph (d), the different letters in the same bar category mean significantly different at a confidence level of 95%.

**FIGURE 6:**
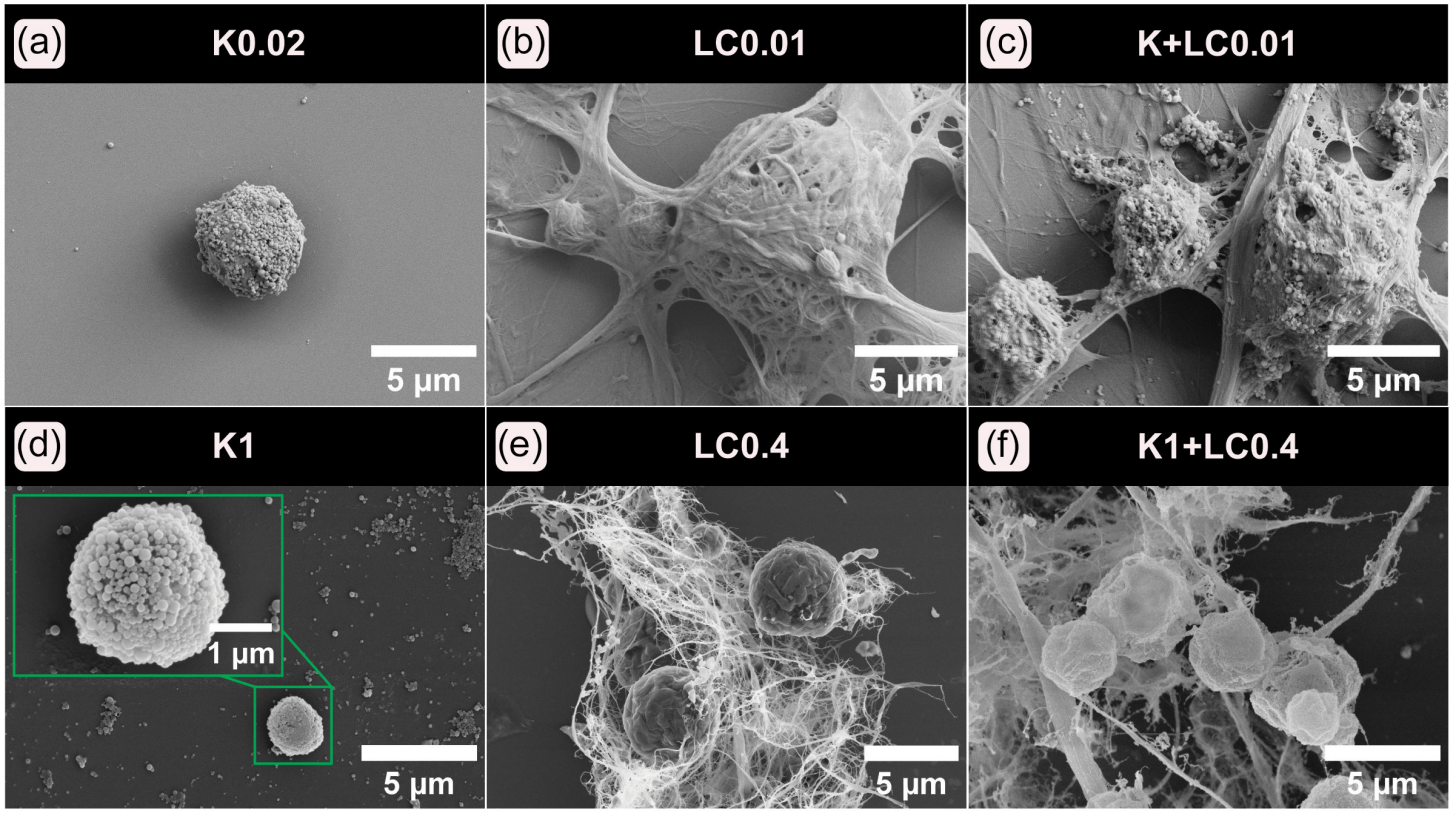
FESEM and cryo‐FESEM of emulsion droplets. FESEM images of PCL Pickering emulsion stabilized by (a) 0.02 wt% K‐LNPs, (b) 0.01 wt% LCNFs, and combination of (c) 0.02 wt% K‐LNPs + 0.01 wt% LCNFs. Cryo‐SEM images of hexadecane Pickering emulsion stabilized by (d) 1 wt% K‐LNPs, (e) 0.4 wt% LCNFs, and combination of (f) 1 wt% K‐LNPs + 0.4 wt% LCNFs.

Droplets at low particle content (K0.02, LC0.01, and K + LC0.01) were examined by FESEM. Furthermore, cryo‐FESEM was used to investigate droplets with high concentrations of particles: K1, LC0.4, and K1 + LC0.4. Both measurements illustrated the different stabilization mechanisms of LNPs and LCNFs. LNPs stabilize emulsions by adsorbing onto individual droplet surfaces, as indicated in Figure 6a,d. In contrast, LCNFs utilize their fibrous structure to form a network that bridges multiple droplets (Figure 6b,e). Meanwhile, when LNPs are mixed concurrently with LCNFs, LNPs predominantly adsorb at the oil–water interfaces. LCNFs may also adsorb at the interface, but it’s most dominating role is the formation of a fibril network in the water phase (Figure 6c,f). Both mechanisms prevent oil droplet coalescence and their combination is the most efficient system. The stabilization mechanism was unaffected by concentration and oil phase types, as cryo‐FESEM at higher concentrations of particles with hexadecane as oil (Figure 6d–f) showed similar phenomena compared with lower particle content with PCL in toluene as the oil phase in FESEM images (Figure 6a–c).

Further synergistic effects of LCNFs and LNPs were observed at the higher LCNF content. Combining 0.05 wt% LCNFs with 0.02 wt% K‐LNPs significantly reduced the droplet size to 44.5 ± 0.1 µm (Figure 5c), producing the smallest droplets among the formulations in this section. At higher fibril concentrations, more fibrils were available to create network formation and prevent oil droplets from coalescence, giving time to the K‐LNPs to reach and adsorb at the oil–water interface. The reduced droplet size correlated directly with the lowest *γ*_s_ (65.2 mN/m) and *γ* *_i_* (24.6 mN/m) of K0.02 + LC0.05 compared to other systems, as presented in Figure 5d. Overall, the micrographs of the emulsion droplets corroborated the DLS data, with smaller droplets observed when a combination of K‐LNPs and LCNFs was used, as shown in Figure 5b. The phase observed at the bottom of the tubes is a water dispersion of nanoparticles with basically no significant presence of oil droplets, as shown in Figure S7. The relationship between droplet size and surface/interfacial tension observed in this study reflects fundamental principles of emulsion formation thermodynamics as demonstrated herein using biobased emulsifiers. Systems with lower *γ* *_i_* require less energy input to generate new surface area, making it thermodynamically more favorable to produce smaller droplets. This synergy effect reflects that combining different natural emulsifiers can enhance stability and properties beyond the capabilities of individual components.

## 3 Conclusions

This study demonstrates that tailoring the interfacial properties of renewable lignocellulosic particles enables controlling the type of emulsion formed. We systematically assessed key variables such as wettability, surface chemistry, and geometry by isolating the dominant variable in each comparison while holding particle size and surface charge constant, thereby minimizing ambiguous attribution of their individual effects. Our systematic analysis involved a rather unique combination of experimental techniques, including electrical conductivity measurements, optical and electron microscopy, and surface sensitive techniques like XPS and surface and interfacial tension measurements. We provide direct evidence, novel for lignocellulose nanoparticles, that emulsion type switched from oil‐in‐water to water‐in‐oil as a function of wettability, regardless of water:oil volume ratio. As evidenced by electrical conductivity, LA‐LNPs generated water‐in‐oil emulsions and K‐LNPs produced oil‐in‐water emulsions, confirming that particle wettability regulates emulsion type. We also confirmed different surface chemistry between K‐LNPs and LA‐LNPs by XPS. The effect of wettability was also verified with fibrillar particles, where residual lignin in LCNFs enhanced interfacial affinity compared to CNFs. Finally, we present that lignocellulosic nanoparticles with tunable geometry and wettability can be combined to drastically tailor Pickering emulsion properties. The combination of 0.01% LCNF and 0.02% LNPs in simultaneous mixing yielded stable emulsions with no oil phase separation at a 2:1 water‐to‐oil ratio. This demonstrates that combining different natural emulsifiers achieves better emulsion stability than either stabilizer alone. Spherical LNPs yielded smaller droplets via close packing of nanoparticles on the droplet surface, while fibrillar LCNFs stabilized larger oil fractions at lower dosage by bridging droplets into a cellulose fibrillar percolating network. The mechanism of stabilization of LNPs, LCNFs, and their synergy were verified by electron microscopy (FESEM and Cryo‐SEM). These findings highlight the potential for tailored renewable lignocellulosic emulsifier systems for industrial applications, tuned by the structural and chemical attributes of the stabilizers. The demand for lignocellulose‐based emulsifiers is rapidly increasing as industries shift toward circular, eco‐friendly practices to address human safety and environmental concerns. Therefore, the findings from this work can have a direct impact on more sustainable applications in cosmetics, advanced materials, environmental technologies, nanomedicine, and other related fields. Water‐in‐oil or oil‐in‐water emulsion formation as a function of wettability and synergy between particles of different geometry could be applied to other particles regardless of the source of material.

## 4 Experimental Section

### 4.1 Materials

Hardwood and Softwood Kraft lignin UPM BioPiva was provided by UPM Biochemicals, Finland. Unbleached paper‐grade softwood pulp having an ISO brightness level of 27% and a kappa number of 29 was sourced from Stora Enso in Finland. Hexadecane, glycerol, diiodomethane, tetrahydrofuran (THF), poly‐L‐Lysine (PLL), polycaprolactone (PCL), and polyethylene imine (PEI) were purchased from Sigma‐Aldrich. Other chemicals used were acetone, hydrochloric acid (HCl) 0.1 M, and sodium hydroxide (NaOH) 0.1 M, purchased from Merck. To investigate the effect of wettability on the formation of Pickering emulsions, we used softwood kraft lignin esterified with lauric acid (reported as LA‐Lignin) with a degree of substitution (DS) of 81% according to quantitative ^31^P NMR analysis. Characterization of this LA‐Lignin is provided in Figure S1.

### 4.2 CNFs and LCNFs

CNFs were prepared following Osterberg et al. [35]. Briefly, never‑dried bleached hardwood Kraft pulp was converted to the sodium form and then mechanically disintegrated. Fibrillation used a Microfluidics M‑110P microfluidizer (Newton, MA, USA): one pass through 400 and 200 μm chambers and then six passes through 400 and 100 μm chambers, at an operating pressure of 2000 bar. The resulting CNF suspension had a solids content of 2 wt%. LCNFs were produced from paper‐grade unbleached softwood pulp using a Hollander beater and an ultra‐fine friction grinder, as previously reported [22]. Briefly, the never‐dried, unbleached paper‐grade pulp fibers were first rinsed with a 0.01 M solution of HCl to remove all metal ions, and then washed with deionized water until the pH reached ~5. After that, the carboxyl groups were converted into their sodium form by treating the fibers with 0.001 M NaHCO_3_ until the pH reached ~7. The fibers were then rinsed with deionized water to remove residual electrolyte until the wash water reached a conductivity below 20 µS/cm. Subsequently, the fibers were fibrillated using a Masuko Super Masscolloider, model MKZB15–50J at 2200 rpm.

### 4.3 LNPs Preparation

Unmodified lignin nanoparticles (K‐LNPs) and lauric acid‐esterified lignin nanoparticles (LA‐LNPs) were prepared via solvent shifting [36]. Both lignins were dried in a vacuum oven at 40 °C overnight before use. The dried kraft lignin was dissolved in acetone:water (3:1 w/w), while lauric acid‐esterified lignin was dissolved in THF to achieve a concentration of 2 wt% in both cases. Each solution was stirred overnight at 250 rpm, and filtered using Whatman GF/F with a pore size of 0.7 µm to remove insoluble lignin. Particles were produced by rapidly pouring 100 g of lignin solution into 350 g of deionized water (DI water) under rapid stirring. The organic solvents were evaporated by rotavapor at 40 °C and 60 mbar, and the dispersion was filtered through Whatman filter grade 1 with pore size 11 µm (particle retention).

### 4.4 Thin Film Preparation

For morphology and contact angle analysis, thin films of LNPs and LCNFs were deposited on silica wafers following established methods [3, 37]. LNP thin films were prepared as follows. PLL was adsorbed onto cleaned silica wafers for 15 min before rinsing. Subsequently, LNPs at a concentration of 0.2 wt% were adsorbed onto the PLL‐coated wafers for a minimum of 45 min. Excess of loosely bound LNPs was removed by rinsing with DI‐water, and the coated wafers were dried with nitrogen gas.

To prepare CNFs and LCNF‐thin films, the CNF and LCNF dispersions were first diluted with DI‐water to 0.2 wt% and dispersed with utrasonicator tip (Branson sonifer S‐450D) for 1 min at 25% amplitude. The CNF and LCNF dispersions were spin‐coated onto PEI‐coated wafers with a Laurell Technologies WS‐650SX‐6NPP/LITE spin coater at 4000 rpm for 1 min.

### 4.5 Preparation of Emulsions

Three types of emulsion were prepared using three different emulsifying systems: LNPs, LCNFs, and a combination of LNPs and LCNFs. In the first stage, we evaluated the effect of the different wettability of particles on Pickering emulsion properties (K‐LNPs vs. LA‐LNPs; and LCNFs vs. CNFs). In the second step, we studied the effect of different geometries on the Pickering emulsion stability. We used K‐LNPs (0.001–1 wt%) and LCNFs (0.001–0.4 wt%) in the Pickering emulsion system. The maximum LCNFs content in the emulsion was 0.4 wt% due to the solid content limitation of LCNFs dispersion. In the last step, we evaluated the synergistic effect of the combination of K‐LNPs (0.02 wt%) and LCNF (0.01–0.05 wt%) on Pickering emulsion characteristics. The emulsions were prepared according to the formulations shown in Table 1 by mixing the Pickering emulsifier and hexadecane (as the oil phase) in one pot using Ultra‐Turrax homogenizer (T18 basic IKA Works GmbH, Germany) at a speed of 10 000 rpm for 2 min [38]. K in the name means that the emulsion was emulsified with K‐LNPs, LA refers to LA‐LNPs, and LC and CN mean LCNFs and CNFs, respectively. Meanwhile, the number following the alphabetic letter indicates particles content in the emulsion system. For instance, K0.001 means an emulsion containing 0.001 wt% of K‐LNPs. The emulsions were further characterized regarding stability, droplet size, conductivity, and structure.

**TABLE 1:** Composition of prepared Pickering emulsions.

Sample	LNPs (dry content: wt%)	LCNFs or CNFs (dry content: wt%)	Water, wt%	**Oil,** ** wt%**	W:O ratio
K0.001	0.001	—	66.7	33.3	2:1
K0.01	0.010	—	66.7	33.3	2:1
K0.02	0.020	—	66.7	33.3	2:1
K0.05	0.050	—	66.6	33.3	2:1
K0.1	0.100	—	66.6	33.3	2:1
K0.2	0.200	—	66.5	33.3	2:1
K0.5	0.500	—	66.3	33.2	2:1
K0.9	0.900	—	66.1	33.0	2:1
K/LA1	1.000	—	66.0	33.0	2:1
LC0.001	—	0.001	66.7	33.3	2:1
LC0.01	—	0.010	66.7	33.3	2:1
LC0.02	—	0.020	66.7	33.3	2:1
LC0.05	—	0.050	66.6	33.3	2:1
CN/LC0.1	—	0.100	66.6	33.3	2:1
LC0.2	—	0.200	66.5	33.3	2:1
LC0.4	—	0.400	66.4	33.2	2:1
K/LA1 (1:1)	1.000	—	49.5	49.5	1:1
K/LA1 (1:2)	1.000	—	33.0	66.0	1:2
K + LC0.01	0.02	0.01	66.6	33.3	2:1
K + LC0.02	0.02	0.02	66.6	33.3	2:1
K + LC0.05	0.02	0.05	66.6	33.3	2:1
K1 + LC0.4	1.000	0.4	66.4	33.2	2:1

*Note:* All percentage values represent weight percentage based on the total emulsion weight. The total volume of emulsion was constant at 13.5 mL.

### 4.6 Particle Characterization

#### 4.6.1 Atomic Force Microscopy (AFM)

The morphology and surface coverage of K‐LNPs, LA‐LNPs, CNFs, and LCNFs thin films were observed using a MultiMode 8 AFM with NanoScope V controller (Bruker) and HQ:NSC15/Al BS probes (MikroMasch‐NanoAndMore, Germany) for tapping mode in air. The acquired images were processed with NanoScope 8.15 or NanoScope Analysis 1.5 software, with only flattening applied for image correction. The root mean square average roughness (Rq) was quantified on images with size of 5 µm^2^. For each sample, we analyzed images from at least two independently prepared thin films.

#### 4.6.2 Particle Size and ζ‐Potential

An Anton Paar Litesizer 500 fitted with a dip cell probe was used to measure the hydrodynamic particle size and ζ‐potential of LNPs, while only ζ‐potential was measured for LCNFs and CNFs. The ζ‐potential values were calculated from the raw electrophoretic mobility data using the Smoluchowski model. The pH of LA‐LNPs was adjusted to obtain a similar ζ‐potential as K‐LNPs, in the range of −35 to −40 mV. For the ζ‐potential of LCNFs and CNFs, the dispersion was measured without pH adjustment. The hydrodynamic diameter (*D*_h_) and ζ‐potential mean values were calculated from three replicates.

#### 4.6.3 Wettability and Surface Free Energy

Static contact angles in air applying the sessile drop technique were used to evaluate the wettability and surface free energy of K‐LNPs and LA‐LNPs. A drop of 5 μL DI water, diiodomethane, or glycerol was applied to the LNPs thin film surface, and pictures were taken every second for 1 minute using a Theta Flex optical tensiometer (Biolin Scientific, Sweden). Similarly, static water contact angle in oil (*θ*_o/w_) was employed to study the wettability of K‐LNPs and LA‐LNPs at the oil–water interface. The thin film was placed with double‐sided tape on the bottom of a quartz crystal chamber, and the chamber was filled with hexadecane. A drop of DI water was placed using a pipette on the top of the film in the oil chamber, and observed with Theta Flex optical tensiometer to calculate *θ*_o/w_. The *θ*_o/w_ is defined as the angle at the three‐phase line (film, oil, and water). Meanwhile, water contact angle in air (film, air, water) is denoted as *θ*_a/w_. The average contact angles were determined from at least two films, measuring two different positions on each film.

Surface free energy of K‐LNPs and LA‐LNPs was calculated by two different methods: Owens–Wendt–Rabel–Kaelble and acid–base using the OneAttension Biolin software [39]. For the Owens–Wendt–Rabel–Kaelble method, the surface free energy was determined based on two liquids (water and diiodomethane) contact angles in air on the substrate (LNPs thin film) using Equation (1):

(1) $$\sqrt{\gamma_{\mathrm{sv}}^{d}\gamma_{\mathrm{lv}}^{d}}+\sqrt{\gamma_{\mathrm{sv}}^{p}\gamma_{\mathrm{lv}}^{p}}=0.5\gamma_{\mathrm{lv}}\left(1+\cos\theta_{\gamma }\right)$$

Meanwhile, the acid–base method, based on contact angles of three liquids (water, glycerol, and diiodomethane) in air was employed using the following Equation (2):

(2) $$\sqrt{\gamma_{\mathrm{sv}}^{d}\gamma_{\mathrm{lv}}^{d}}+\sqrt{\gamma_{\mathrm{sv}}^{\mathrm{acid}}\gamma_{\mathrm{lv}}^{\mathrm{base}}}+\sqrt{\gamma_{\mathrm{sv}}^{\mathrm{base}}\gamma_{\mathrm{lv}}^{\mathrm{acid}}}=0.5\gamma_{\mathrm{lv}}\left(1+\cos\theta_{\gamma }\right)$$

where *θ*_γ_ is the liquid contact angle on the substrate, *γ*_lv_ is surface tension of liquid, $\gamma_{\mathrm{lv}}^{d}$ is the dispersive component of liquid, $\gamma_{\mathrm{lv}}^{\mathrm{base}}$ is base component of liquid, $\gamma_{\mathrm{lv}}^{\mathrm{acid}}$is acid component of liquid, $\gamma_{\mathrm{lv}}^{p}$ is the polar component of liquid, $\gamma_{\mathrm{sv}}^{d}$ is the dispersive component of the substrate, $\gamma_{\mathrm{sv}}^{p}$ is the polar component of the substrate, $\gamma_{\mathrm{sv}}^{\mathrm{acid}}$is acid component of substrate, and $\gamma_{\mathrm{sv}}^{\mathrm{base}}$ is base component of substrate.

#### 4.6.4 XPS

The surface chemistry of bulk lignins and nanoparticles, both for K‐lignin and LA‐lignin, was analyzed by a Kratos AXIS Ultra DLD XPS using a monochromated Al Kα X‐ray source (1486.7 eV) run at 100 W. In this study, bulk kraft lignin was denoted as K‐lignin, while its nanoparticle was described as K‐LNPs. Bulk lauric acid lignin and its nanoparticles were respectively called as LA‐lignin and LA‐LNPs. For nanoparticles, LNPs were freeze‐dried for 24 h using a Labconco FreeZone 2.5 Freeze Dryer before analysis by XPS. A pass energy of 80 eV and a step size of 1.0 eV were used for survey spectra, while a pass energy of 20 eV and a step size of 0.1 eV were used for high‐resolution spectra. Photoelectrons were collected at a 90° take‐off angle under ultra‐high vacuum conditions, with a base pressure typically below 1 × 10^−9^ Torr. Both survey and high‐resolution spectra were collected from three different spots on each sample surface and average values were calculated using the results from each position. All spectra were charge‐corrected relative to the position of C─C carbon bonds at 284.8 eV. Peak fitting of the C 1s and O 1s regions was done according to standard chemical shifts [40] using Shirley backgrounds and Gaussian–Lorentzian peak shapes with the same full‐width‐at‐half‐maximum for all peaks from the same element. The CasaXPS software was used for all data analysis.

### 4.7 Emulsion Characterization Methods

#### 4.7.1 Emulsion Stability, Droplet Size Analysis, and Conductivity Measurement

Emulsion stability was evaluated after 0 h, 1 day, 5 days, and 7 days of storage. Physical stability was assessed using photographic documentation. The excess oil fraction after 1 day of storage was calculated as the percentage of excess oil in the total system. The microstructure of the droplets during 1 week of emulsion storage was assessed by optical microscopy using an Olympus BX53M microscope equipped with cellSens imaging software. The emulsion was diluted in water 10–50 times before being applied to a microscope slide, and the droplet images were captured at 20 times magnification. The optical images of emulsions containing K‐LNPs and LA‐LNPs 1 wt% with different W:O ratio (K1 1:1, K1 1:2, K1 2:1, LA1 1:1, LA1 1:2, and LA1 2:1) were used to calculate the mean droplet diameter by ImageJ. In addition, the dispersion of LCNFs and CNFs prepared in Section 4.2 was also assessed with optical microscopy to evaluate the coarse fibrils.

Emulsion droplet size was also analyzed using dynamic light scattering (DLS) Mastersizer 2000 (Malvern, UK) at a refractive index of 1.49 to determine area‐weighted (Sauter) mean droplet diameters (D [2, 3], Equation (3)):

(3) $$D\;\left[3,2\right]=\;\frac{\text{Σ}n_{i}d_{i}^{3}}{\text{Σ}n_{i}d_{i}^{2}}$$

$n_{i}$ and $d_{i}$ represent the number and the diameter of particulate matter.

In LA‐LNPs series, the droplet size was calculated only from optical microscopy images using imageJ because the water‐in‐oil emulsion could not be dispersed in water for DLS analysis.

The conductivity of emulsions K1 (1:2) and L1 (1:2) was measured by Mettler Toledo FiveEasy Plus conductometer. The excess non‐emulsified oil phase was first removed, and the sensor was immersed in the emulsion phase.

#### 4.7.2 Surface and Interfacial Tension

The du Noüy ring method (KSV Sigma 70, Finland) was applied to assess how Pickering emulsifier dispersion with different concentration affects surface and interfacial tension of water at room temperature [41]. A platinum wire ring was submerged in the LNPs dispersion, and the net force exerted on the ring during withdrawal was recorded. The experiments were conducted at a speed up of 5 mm/min, speed down of 20 mm/min, and dwell down of 5%. The surface tension (*γ*_s_) was calculated in Sigma software from the required force to raise the ring from the LNPs or LCNFs dispersion to air. Meanwhile, interfacial tension (*γ* *_i_*) was measured by the force to raise the ring from LNPs or LCNFs dispersion to oil (hexadecane). The reported *γ*_s_ and *γ* *_i_* values represent averages from three replicate measurements, with 5 data points collected per measurement. Furthermore, statistical analysis using ANOVA followed by the Duncan test was done to observe the significant effect of Pickering emulsifier on reducing the surface and interfacial tension of water.

#### 4.7.3 Field Emission Scanning Electron Microscopy (FESEM) and Cryo‐FESEM

FESEM and Cryo‐FESEM were used to investigate emulsion droplet and bridging phenomena due to LCNF addition. For FESEM images, hexadecane was replaced with 10% PCL in toluene to create oil droplets that could be dried. We selected 10% PCL in toluene because both toluene (27 mN m^−1^) and hexadecane (28.5 mN m^−1^) have similar surface tensions [42]. Emulsions were prepared from K‐LNPs (K0.02), LCNF (LC0.01), and the combination of K‐LNPs and LCNF (K + LC0.01). The emulsions were diluted five times with DI water, and they were applied to silica wafers. The coated wafers were dried in a vacuum oven at 40 °C overnight. Prior to SEM imaging, dried emulsions on silica wafer were coated with 10 nm of gold‐palladium alloy in Leica EM ACE600. SEM images were recorded with a SEM Zeiss Sigma VP (Germany) at 5 kV with 500–20 000 times magnifications.

We further investigated the network formation between the droplets by cryo‐SEM (Aquilos 2, Thermo Fisher Scientific). An emulsion with hexadecane as the oil phase was diluted 10–20 times with DI water, and 10 µl of diluted emulsion was dropped on glass slides. The drop of emulsion was swabbed to obtain a flat surface of emulsion. Subsequently, the sample was placed in the cryo‐SEM chamber with a temperature of −50 °C. Before cryo‐SEM imaging, the emulsion was coated with platinum for 5 nm. Images were recorded at 2 kV with a magnification ranging from 15 000 to 100 000 times.

## Author Contributions

**Nissa Nurfajrin Solihat**: conceptualization, data curation, formal analysis, investigation, methodology, validation, and writing – original draft. **Muhammad Farooq**: conceptualization, visualization, writing – review and editing. **Juan José Valle‐Delgado**: conceptualization, writing – review and editing. **Elijah Mark Garcia**: data curation and formal analysis (lignin modification). **Kristoffer Meinander**: data curation and formal analysis (XPS). **Monika Österberg**: conceptualization, writing – review and editing, resources, supervision, and funding acquisition. The manuscript was written through the contributions of all authors. All authors have approved the final version of the manuscript.

## Funding

This study was supported by the Lembaga Pengelola Dana Pendidikan (SKPB6412/LPDP/LPDP.3/2023) and the Academy of Finland (345553, 345556).

## Conflicts of Interest

The authors declare no conflicts of interest.

## Supporting information

Supplementary Material

## Data Availability

The data that support the findings of this study are available on request from the corresponding author.
