# Supplementary material for: Oil‐In‐Water or Water‐In‐Oil Pickering Emulsion: Role of Lignocellulose‐Based Nanoparticle Wettability and Geometry
Source: Small Sci. 2026 Nov 11;6(11):e70437. doi: 10.1002/smsc.70437 (PMC13753703; doi:10.1002/smsc.70437)
Supplement: Supplementary file 1 [file SMSC-6-e70437-s001.pdf]

Supporting Information

Oil-in-water or water-in-oil Pickering emulsion: role of lignocellulose-based nanoparticle wettability and geometry

*Nissa Nurfajrin Solihat[1], Muhammad Farooq[1], Juan José Valle-Delgado[1], Elijah Mark Garcia[1], Kristoffer Meinander[1], Monika Österberg[1]**

[1]Department of Bioproducts and Biosystems, School of Chemical Engineering, Aalto University, Espoo, Finland.

Spectroscopic analyses of the lignins

The sample preparation and parameters for determining the functional groups and the hydroxyl content of the unmodified softwood kraft lignin and the lauric acid-esterified lignin were described in our previous studies [1, 2]. The Fourier transform infrared (FTIR) spectra were recorded via Spectrum Two FTIR spectrometer (Perkin-Elmer) with an attenuated total reflectance (ATR) module. The phosphorus nuclear magnetic resonance (^{31}P NMR) spectra were acquired by Bruker NMR Spectrometer AV III 400 MHz. Quantification was performed with the use of *N*-hydroxy-5-norbornene-2,3-dicarboxylic acid imine (NHND) as an internal standard.

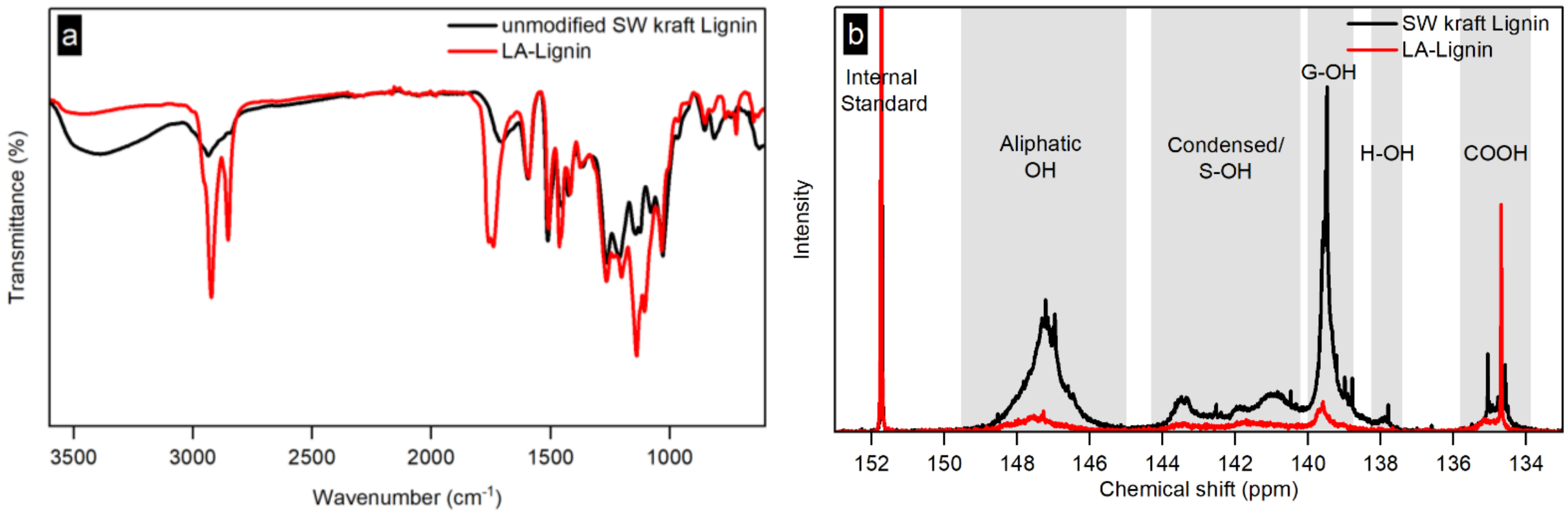

Figure S1. Characterization of the lauric acid-esterified lignin (La-Lignin) and the unmodified SW kraft lignin via (a) FTIR spectroscopy and (b) quantitative ^{31}P NMR analysis.

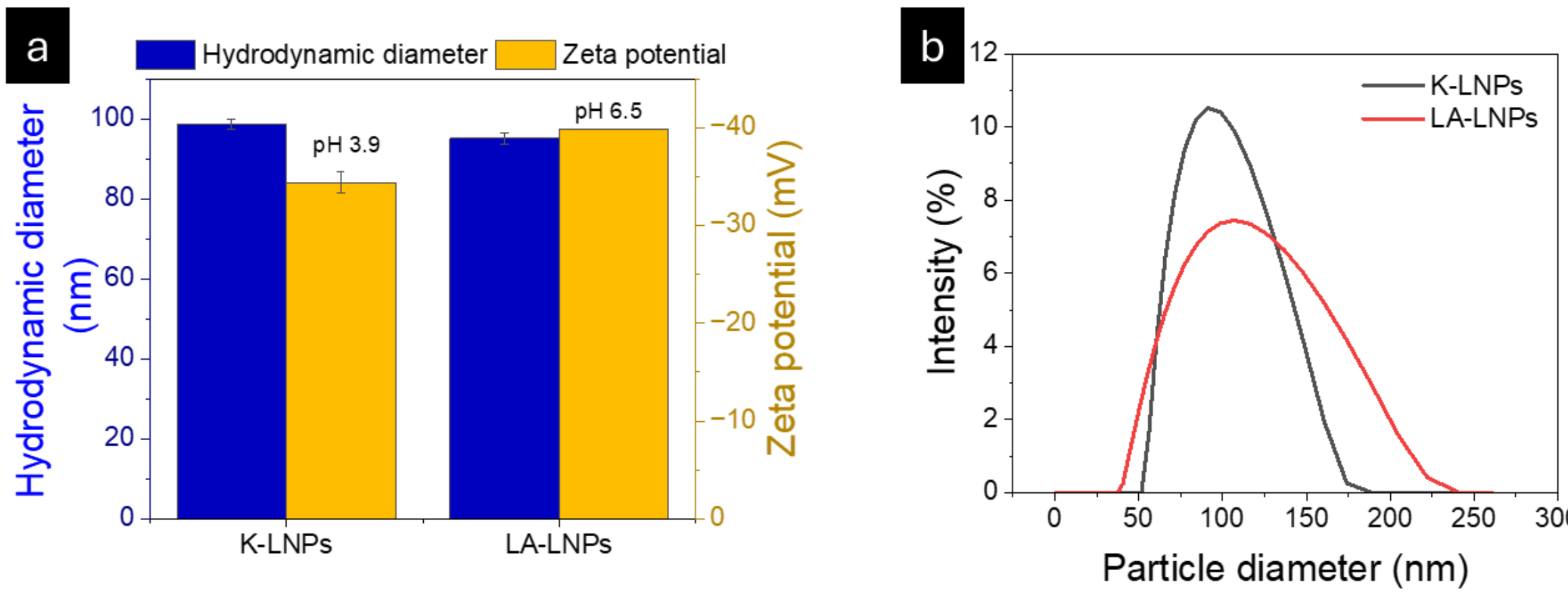

Figure S2. (a) Hydrodynamic diameter, ζ-potential, and (b) particle size distribution of K-LNPs and LA-LNPs from dynamic light scattering (DLS) measurement.

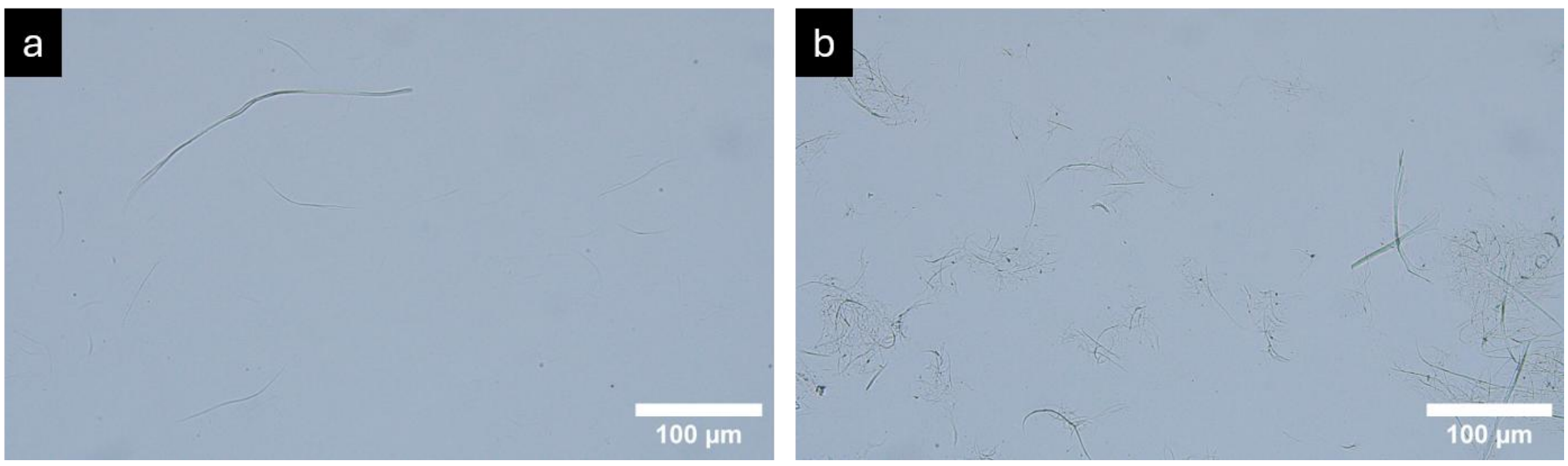

Figure S3. Optical microscopy of (a) CNFs and (b) LCNFs dispersion 0.2 %wt.

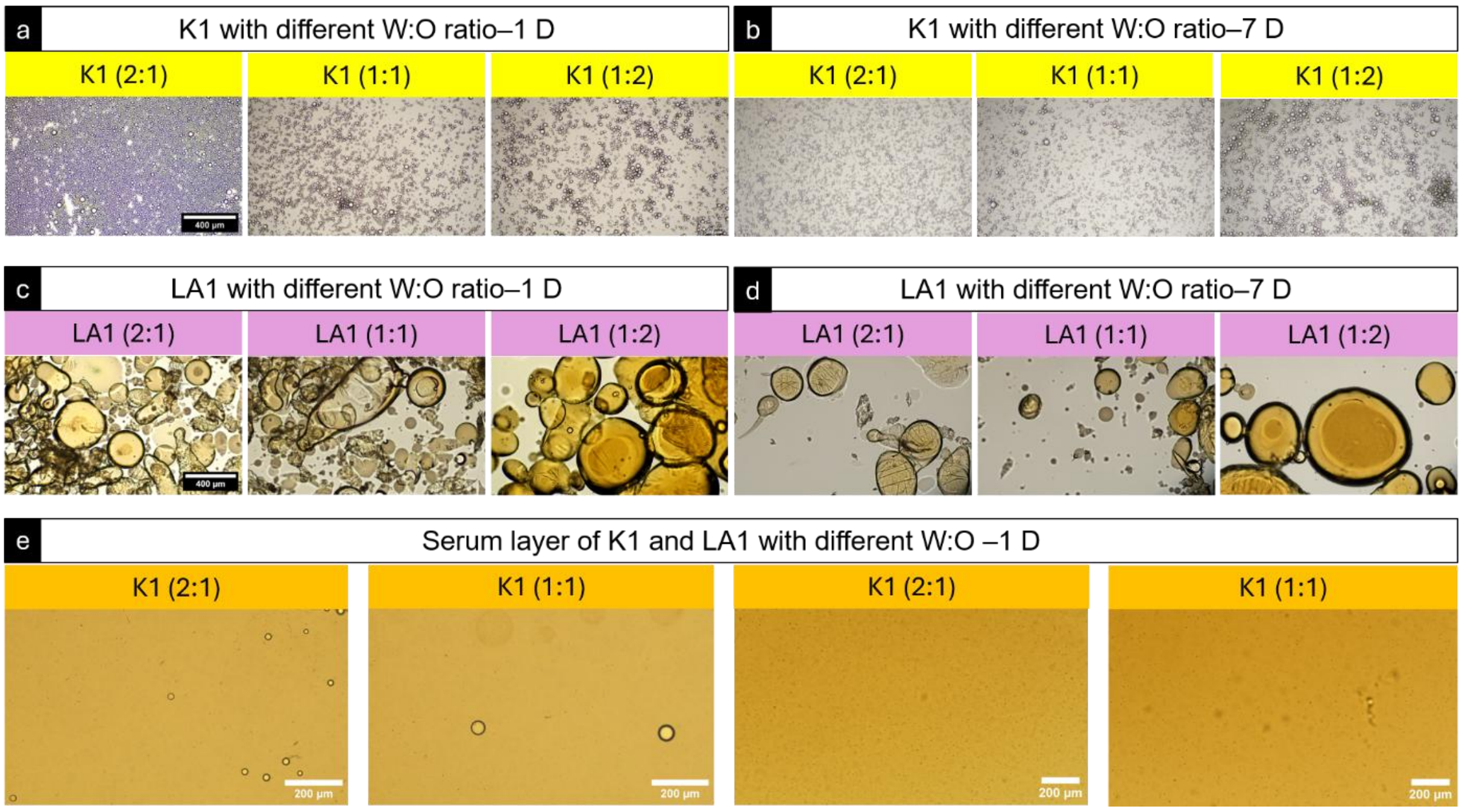

Figure S4. Optical images of (a-b) diluted oil-in-water emulsion layer K1 and (c-d) water-in-oil emulsion LA1 with different W:O ratio (2:1 – 1:2) after one and seven days of emulsification. All the scales for optical microscopy images in Figure a to d are the same, 400 µm.(e) Optical images of bottom layer (undiluted aqueous nanoparticle dispersion) in the tube of K1 and L1 with W:O 2:1 and 1:1 after one day of emulsification

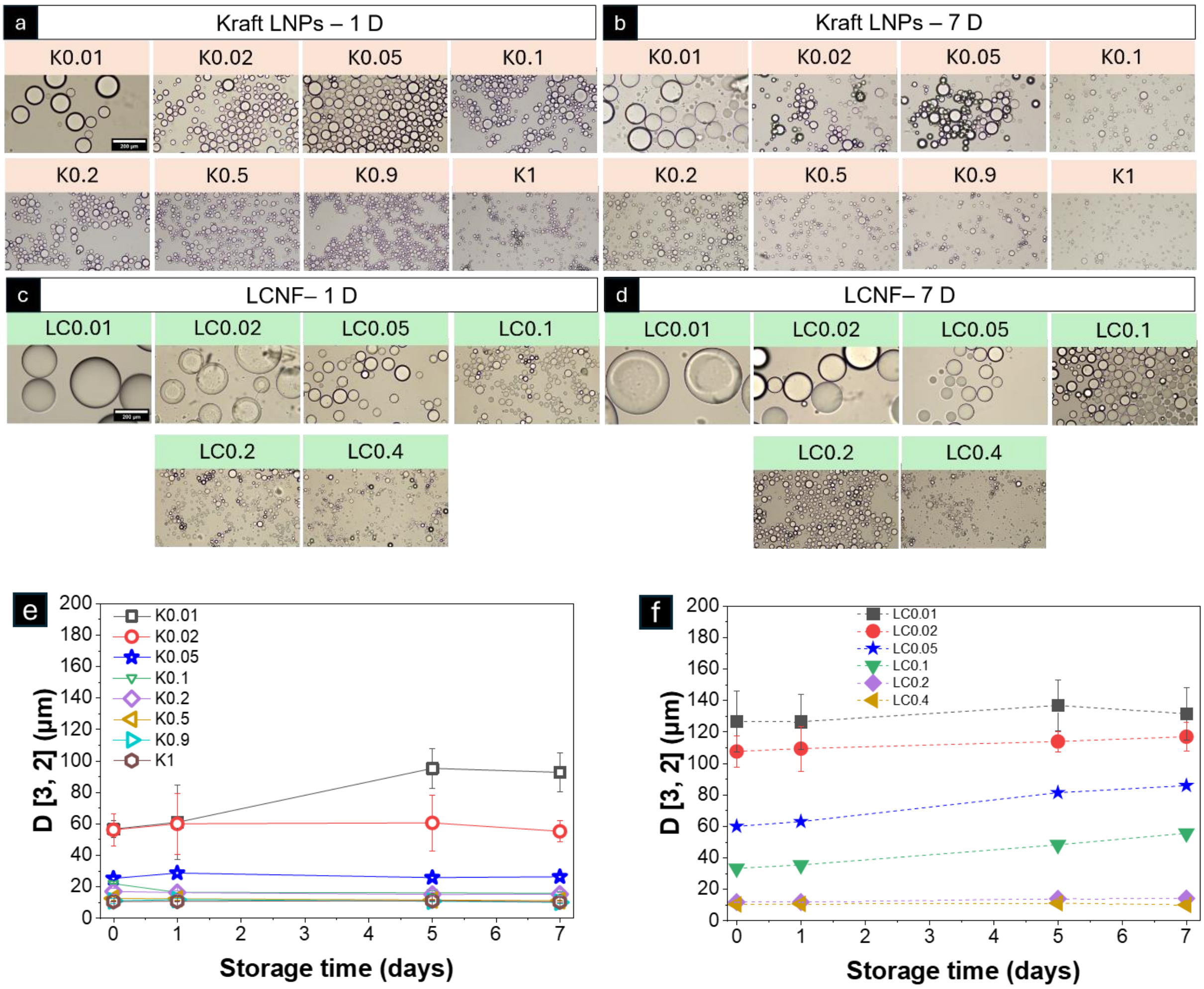

Figure S5. Droplet images by optical microscopy of emulsion stabilised by (a-b) LNPs and (c-d) LCNF after one and seven day storage. Effect of particle content and storage time up to seven days on droplet size of emulsion containing (e) K-LNPs and (f) LCNF. All the scales for optical microscopy images are the same, 200 μm.

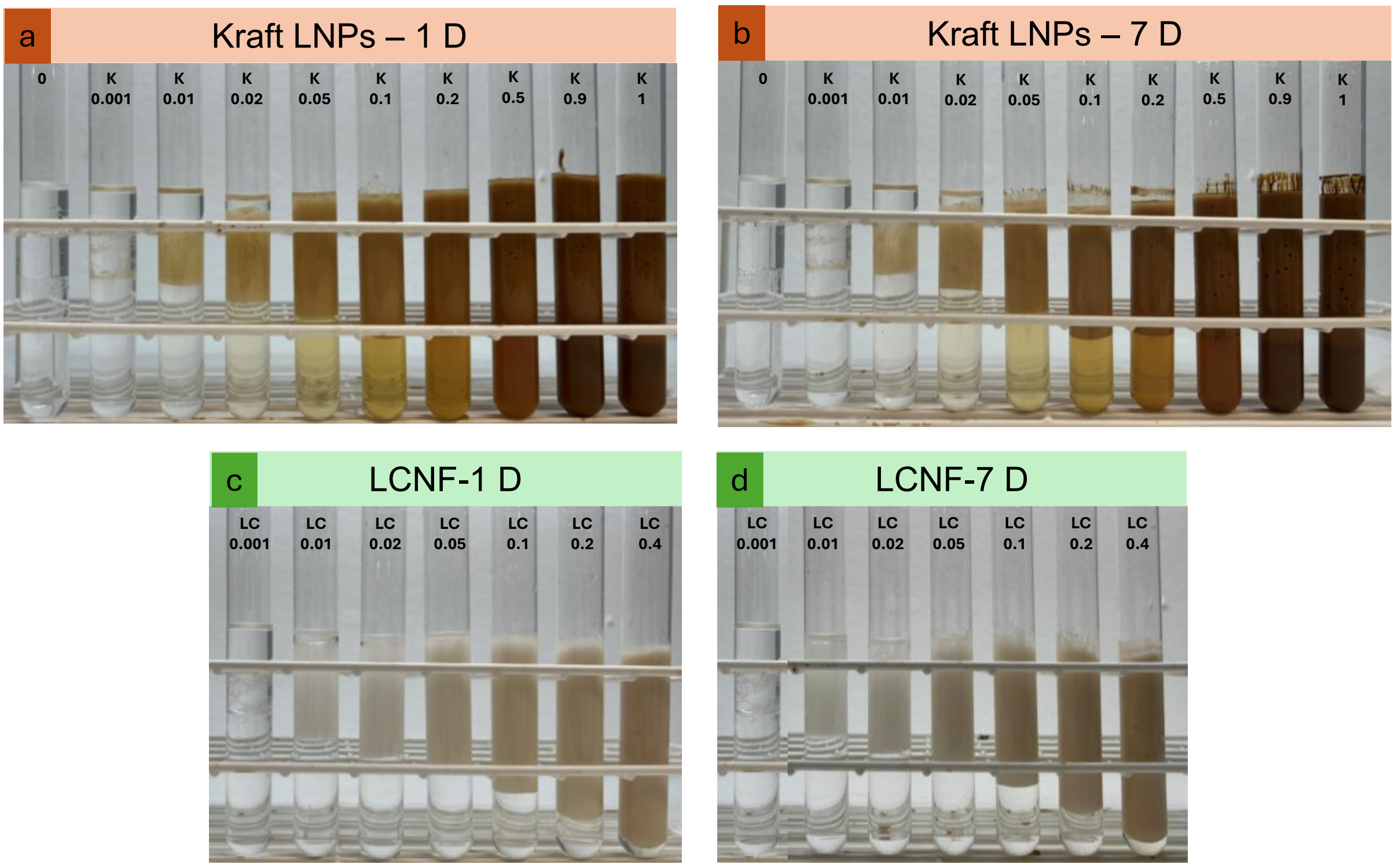

Figure S6. Visual assessment of emulsion stabilized by (a-b) LNPs and (c-d) LCNFs after one and seven days of storage.

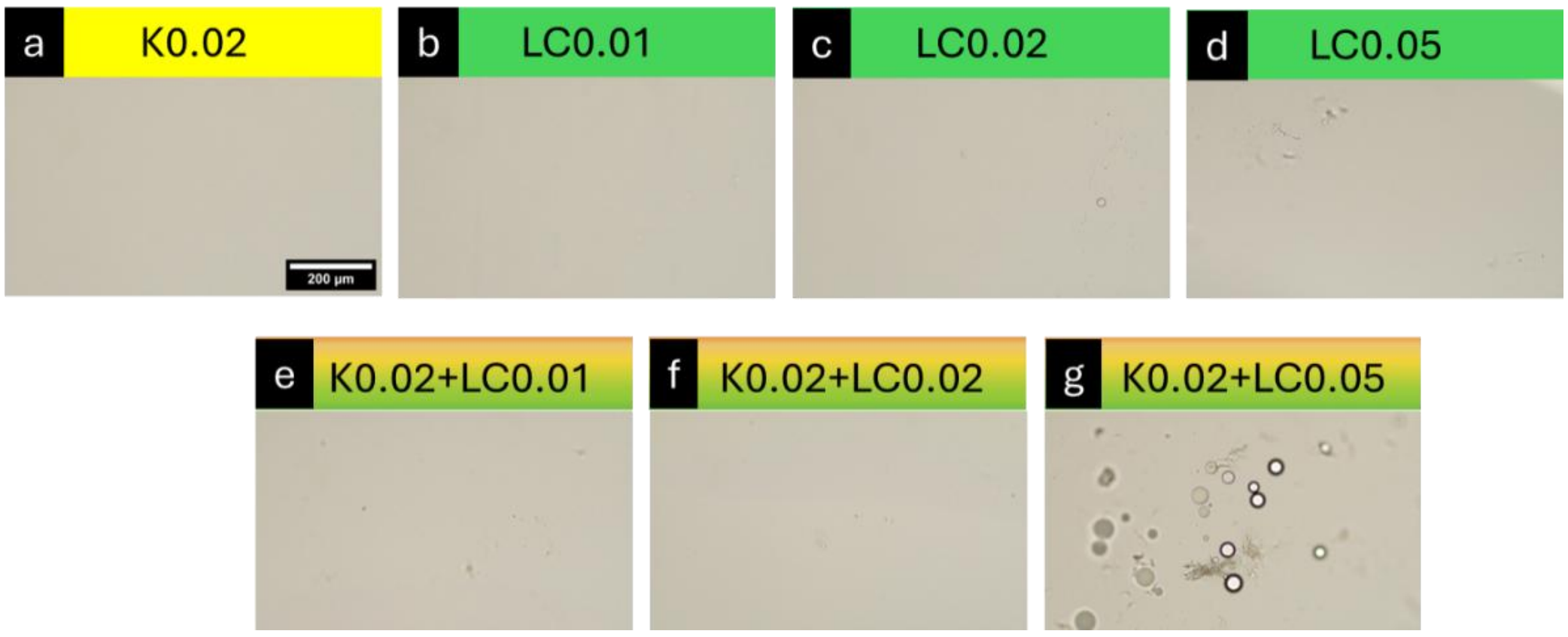

Figure S7. Optical microscopy of bottom layer (aqueous nanoparticle dispersion) in the tube of (a) K0.01, (b) LC.0.01, (c) LC0.02, (d) LC0.05, (e) K0.02+LC0.01, (f) K0.02+LC0.02, (g) K0.02+LC0.05

XPS discussion

In bulk sample, LA-lignin has a lower oxygen content (14.4%) than K-lignin (21.1%). In detail, the O 1s region showed a lower C-O bond in LA-lignin (69.9%) compared to K-lignin (88.1%), while the

proportion of O in carbonyl (C=O) was higher, 28.6% for LA-lignin and 9.9% for K-lignin. These trends suggest that hydroxyl groups in LA lignin were esterified with lauric acid. The introduction of lauric acid in LA-lignin also caused a higher carbon content in LA-lignin (84.8%) compared to K-lignin (77.8%). The C 1s region showed a high proportion C–C in LA-lignin, likely corresponding to the alkyl chain of lauric acid. Additionally, the O–C=O was also more abundant in LA-lignin (LA-lignin 2.9% and K-lignin 1.9%), which indicated esterification had happened in LA-lignin. The detailed atomic percentage of each element is shown in **Table S1**. In summary, counterintuitive and more significant differences in surface chemistry between bulk and LNPs were observed for the LA-lignin due to the introduction of lauric acid into the lignin structure.

Table S1. XPS data for bulk and particles of modified (LA) and native lignin (K)

**Element/Peak**	**Atomic fraction (%)**			
	**LA-lignin bulk**	**LA-LNPs**	**K-lignin bulk**	**K-LNPs**
C 1s	84.8 ± 0.1	86.1 ± 0.2	77.8 ± 0.0	74.2 ± 0.6
O 1s	14.4 ± 0.2	13.1 ± 0.2	21.1 ± 0.0	23.9 ± 0.5
N 1s	0.4 ± 0.1	0.4 ± 0.2	0.1 ± 0.0	0.1 ± 0.0
S 2p	0.4 ± 0.0	0.3 ± 0.0	1.0 ± 0.0	1.5 ± 20.1
Total element	*100.0*	*100.0*	*100.0*	*100.0*
O/C ratio	0.17	0.15	0.27	0.32
**C 1s**				
C (ar-C)	7.8 ± 0.3	2.8 ± 0.3	9.1 ± 0.7	9.7 ± 0.4
C (C–C)	67.6 ± 0.1	75.2 ± 0.3	52.4 ± 0.6	46.9 ± 1.5
C (C–O)	20.8 ± 0.1	18.5 ± 0.3	33.5 ± 0.2	36.2 ± 0.13
C (C=O)	0.9 ± 0.2	0.9 ± 0.1	3.1 ± 0.0	4.6 ± 0.1
C (O–C=O)	2.9 ± 0.0	2.6 ± 0.1	1.9 ± 0.1	2.6 ± 0.1
Total C 1s	*100.0*	*100.0*	*100.0*	*100.0*
**O 1s**				
O (C=O)	28.6 ± 0.1	29.6 ± 0.2	9.9 ± 0.1	11.3 ± 0.5
O (C–O)	69.9 ± 0.1	69.4 ± 0.1	88.1 ± 0.2	87.2 ± 1.9
O (OH)	1.5 ± 0.0	1.0 ± 0.0	2.0 ± 0.1	1.6 ± 2.5
Total O 1s	*100.0*	*100.0*	*100.0*	*100.0*

Table S2. Surface free energy of K-LNPs, LA-LNPs, LCNFs, and CNFs calculated from Owens-Wendt-Rabel-Kaelble and acid-base method

**Method**	**Sample name**	γ^{tot} **[mN/m]**	γ^{d} **[mN/m]**	γ^{p} **[mN/m]**	γ^{acid} **[mN/m]**	γ^{base} **[mN/m]**
Owens-Wendt-Rabel-Kaelble	K-LNPs	76.8 ± 0.3	50.1 ± 0.6	26.6 ± 0.3	-	-
	LA-LNPs	53.8 ± 4.6	38.2 ± 2.9	15.6 ± 2.0	-	-
	LCNFs	73.8 ± 0.6	48.6 ± 0.4	25.2 ± 1.0	-	-
	CNFs	78.7 ± 0.3	48.2 ± 0.4	30.4 ± 0.4	-	-
Acid-base	K-LNPs	66.2 ± 0.2	49.8 ± 0.6	16.3 ± 0.5	1.4 ± 0.0	5.9 ± 0.0
	LA-LNPs	51.2 ± 4.8	38.1 ± 4.2	13.1 ± 0.7	1.9 ± 0.6	3.6 ± 1.1
	LCNFs	64.2 ± 0.4	48.6 ± 0.5	15.6 ± 1.3	1.4 ± 0.1	5.7 ± 0.2
	CNFs	64.7 ± 0.4	48.2 ± 0.5	16.5 ± 0.8	1.3 ± 0.1	6.5 ± 0.0

Table S3. Diameter of droples emulsion stabilized by K-LNPs 1 wt% and LA-LNPs 1wt% with different W:O ratio. The droplets after 1 and 7 days emulsification were measured by Mastersizer (DLS) and imageJ from optical images.

**Sample code**	**Diameter of droplets (µm)**			
	**DLS**		**Microscopy**	
	**D1**	**D7**	**D1**	**D7**
K1 (2:1)	10.7±0.3	10.4±0.7	8.7±0.9	8±1.1
K1 (1:1)	11.7±0.8	10.4±0.2	7.9±0.9	8.6±0.1
K1 (1:2)	12.1±0.7	11.2±0.1	9.3±0.5	9.2±0.1
LA1 (2:1)	NA	NA	238 ± 40	251 ± 34
LA1 (1:1)	NA	NA	222 ± 87	210 ± 19
LA1 (1:2)	NA	NA	322 ± 37	371 ± 97

NA means not available because oil as continuous phase was not suitable for DLS measurement

Table S4. Layer of emulsion based on visual assessment after one day of storage

Code	Excess oil layer(%)	Emulsion layer (%)	Excess water layer (%)
K0.001	37.0	2.7	60.3
K0.01	18.4	22.6	59.1
K0.02	9.3	41.3	49.3
K0.05	2.6	53.3	44.0
K0.1	2.8	61.7	35.5
K0.2	1.2	65.1	33.8
K0.5	1.2	66.8	32.0
K0.9	0.9	69.4	29.8
K1	0.0	75.1	24.9
LC0.001	34.6	7.7	57.7
LC0.01	8.8	42.6	48.7
LC0.02	3.8	51.3	44.9
LC0.05	1.7	59.8	38.5
LC0.1	0.0	79.2	20.8
LC0.2	0.0	81.8	18.2
LC0.4	0.0	93.8	6.2
CN0.1	5.5	74.0	20.5
LA1	38.0	6.3	55.7
K1 (1:1)	0.0	89.3	10.7
K1 (1:2)	4.9	95.1	0.0
LA1 (1:1)	44.1	22.9	33.0
LA1 (1:2)	68.4	31.6	0.0
K + LC0.01	0.0	53.7	46.3
K + LC0.02	0.0	59.2	40.8
K + LC0.05	0.0	62.7	37.3

References

[1] E.M. Garcia, P. Nousiainen, U. Hyväkkö, M. Österberg, Solventless Production of Thermoplastic Lignin Esters for Polymer Blends and Elastomers via Reactive Extrusion, ACS Sustainable Chemistry & Engineering 13 (2025) 12198–209.10.1021/acssuschemeng.5c04676

[2] S. Babaeipour, P. Nousiainen, E. Kimiaei, J. Tienaho, N. Kohlhuber, R. Korpinen, K. Kaipanen, M. Österberg, Thin multifunctional coatings for textiles based on the layer-by-layer application of polyaromatic hybrid nanoparticles, Materials Advances 5 (2024) 6114–31.10.1039/d4ma00085d
